# Supplementary material for: The DNA methylation landscape of primary triple-negative breast cancer
Source: Nat Commun. 2025 Mar 28;16:3041. doi: 10.1038/s41467-025-58158-x (PMC11953470; doi:10.1038/s41467-025-58158-x)
Supplement: Supplementary file 1 — Supplementary Information [file 41467_2025_58158_MOESM1_ESM.pdf]

Supplementary Figure S1

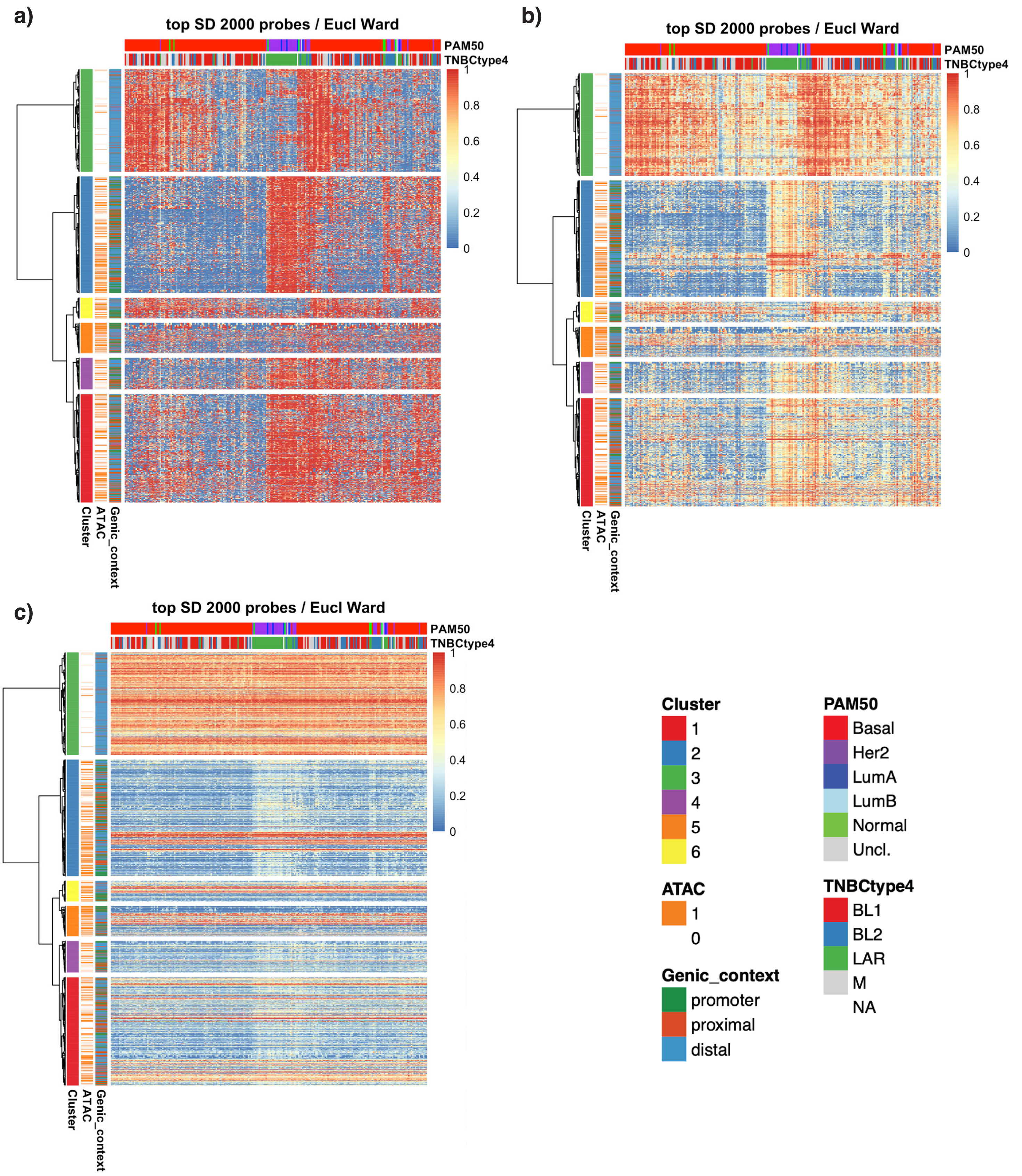

Supplementary Figure S1. Effects of tumor purity adjustment on beta values in 235 SCAN-B TNBC tumors. In panels a-c the 2000 most variant CpGs (by standard deviation, SD) in tumor purity-adjusted beta data were selected. Clustering of CpGs (rows) and samples (columns) is based on tumor purity-adjusted beta values and ordered similarly throughout all panels. **(a)** Tumor purity-adjusted beta values based on the method by Staaf and Aine, PLoS One, 2022. **(b)** Unadjusted, original tumor beta values. **(c)** Inferred normal background beta values from the method by Staaf and Aine, PLoS One, 2022.

Supplementary Figure S2

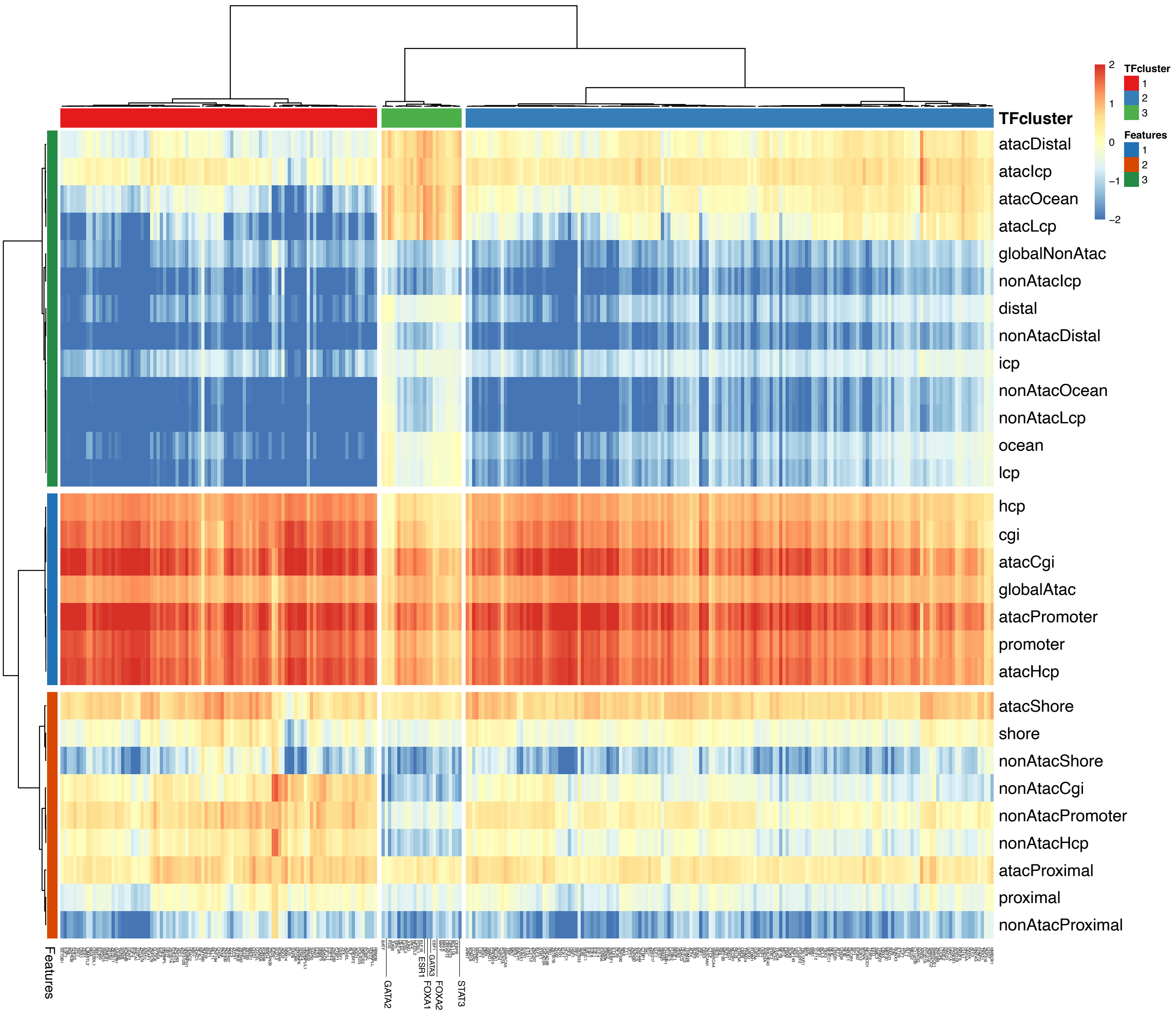

Supplementary Figure S2. Transcription factor (TF) site enrichment by genomic context. 741145 cg-labelled probes were mapped to transcription factor binding sites (TFBSs) using ENCODE data for 340 transcription factors (TFs) derived from 130 cell lines. For each transcription factor (columns), enrichment was calculated as the proportion of CpGs belonging to a specific genomic context (rows) from all CpGs connected to that TF divided by the proportion of CpGs belonging to that genomic context from all CpGs connected to any TF except that one. Hierarchical clustering was performed using the Ward.D method and the resulting trees were cut into 3 groups. Specific TFs are highlighted.

Supplementary Figure S3

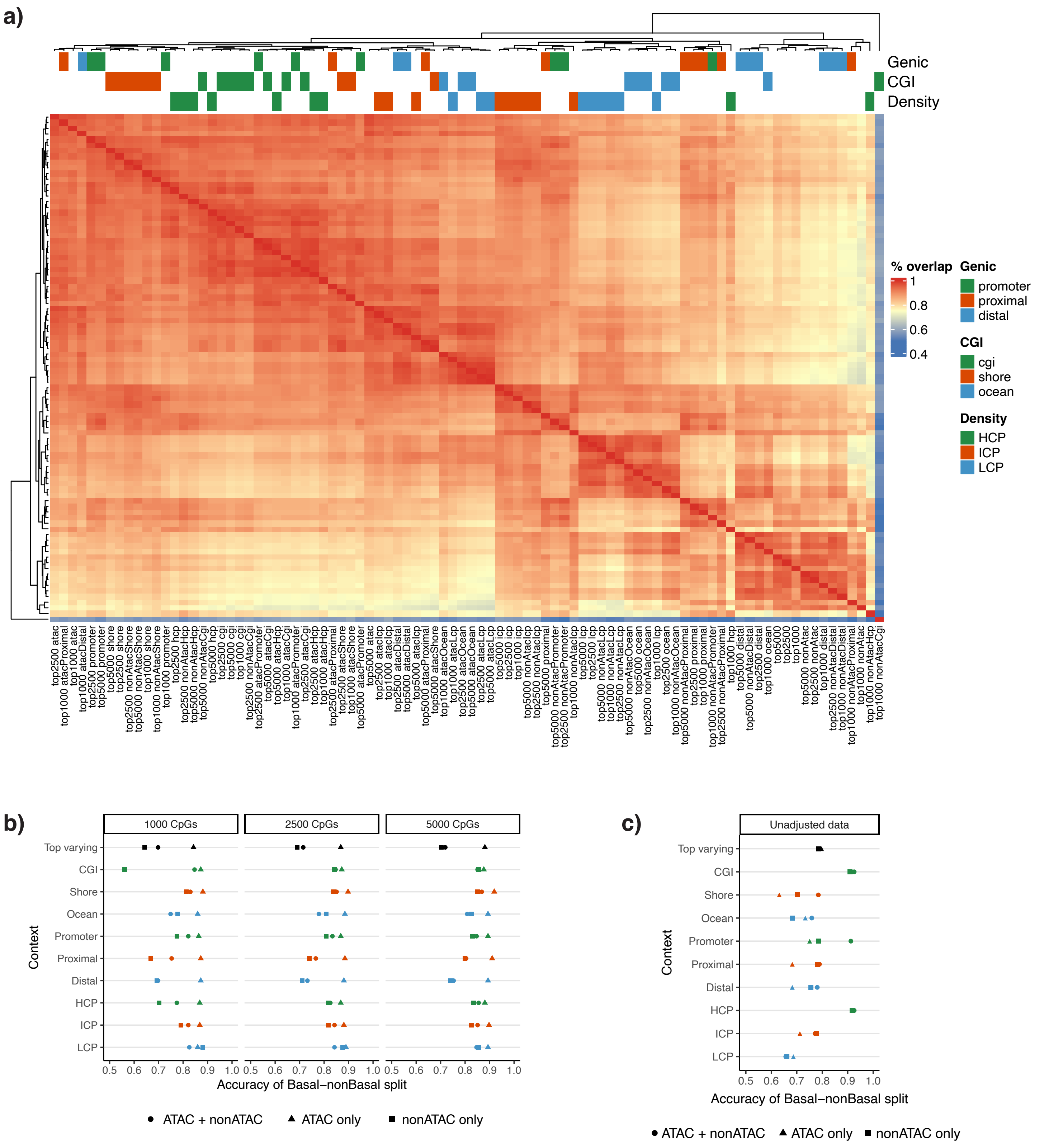

Supplementary Figure S3. CpG contexts and NMF clustering. (a) Two-group (k=2) NMF clustering agreement (as percentage of samples in the same clusters) across classifications using different CpG contexts and CpG numbers. Red means high agreement, blue low agreement. Columns and rows follow the same order. **(b)** Agreement plot between NMF k=2 cluster solutions and PAM50 Basal/nonBasal mRNA subtypes for different CpG contexts and ATAC status using the 1000, 2500, or 5000 most variant CpGs in each data set. **(c)** Same plot as in (b) but using the 5000 most variant CpGs from original, unadjusted beta values. In panels a-b, analyses were performed on tumor cell content-adjusted beta values.

Supplementary Figure S4

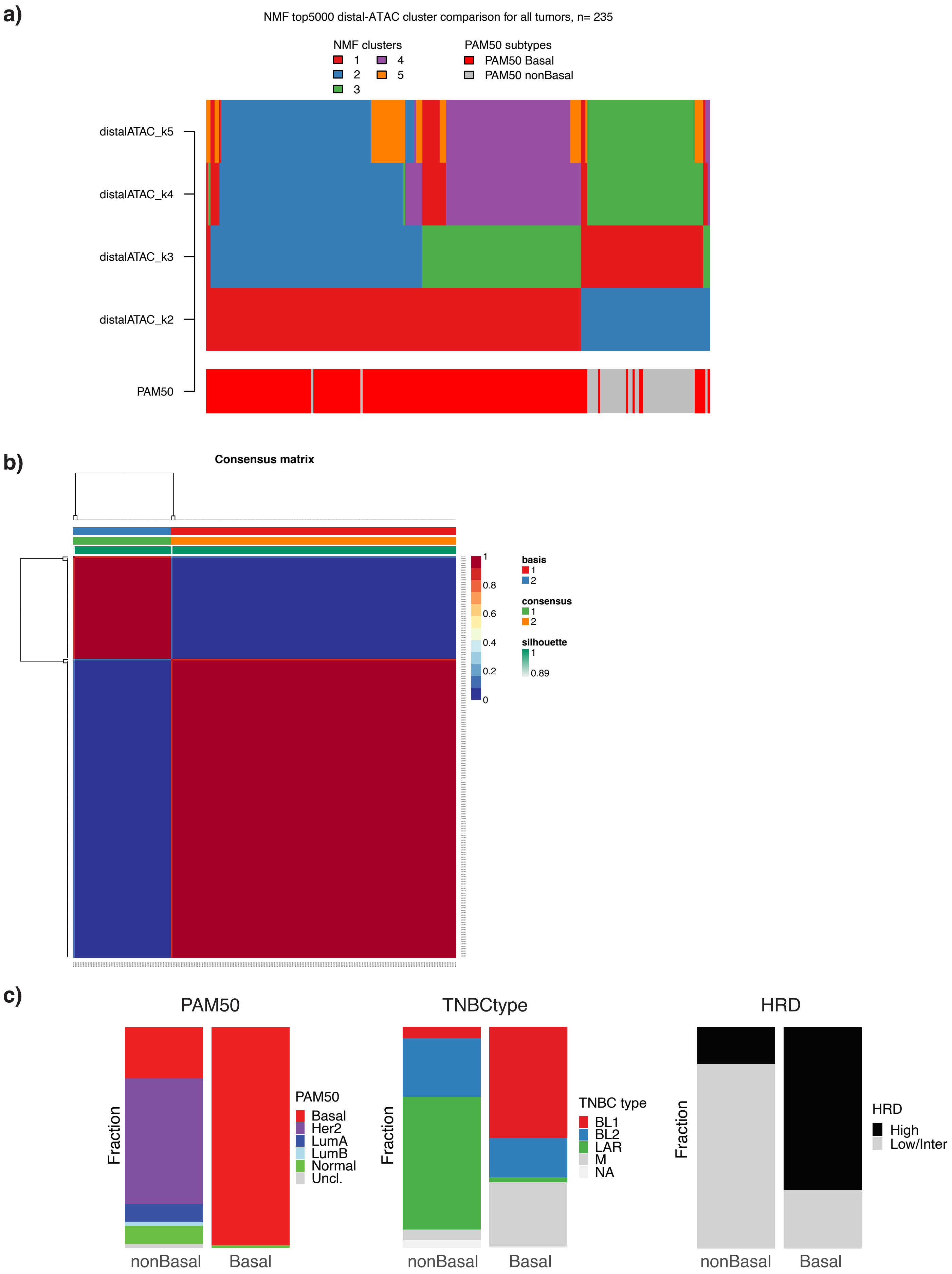

Supplementary Figure S4

d)

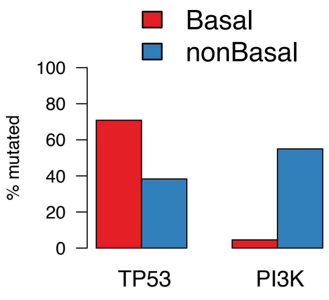

e)

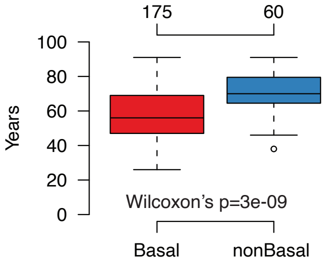

f)

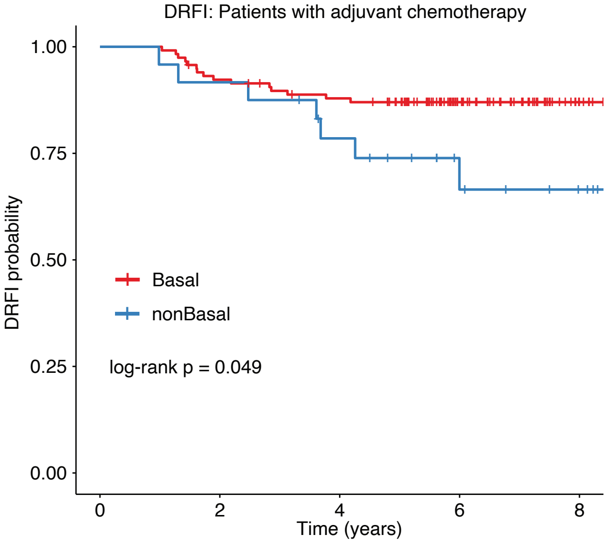

g)

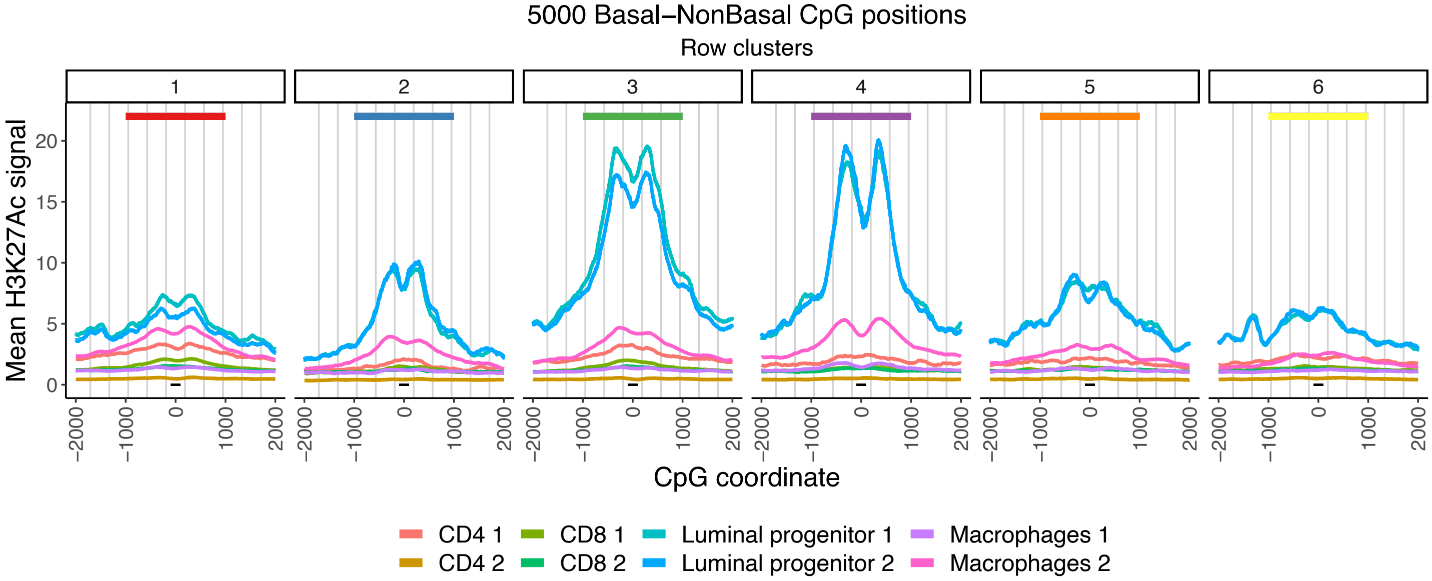

Supplementary Figure S4

h)

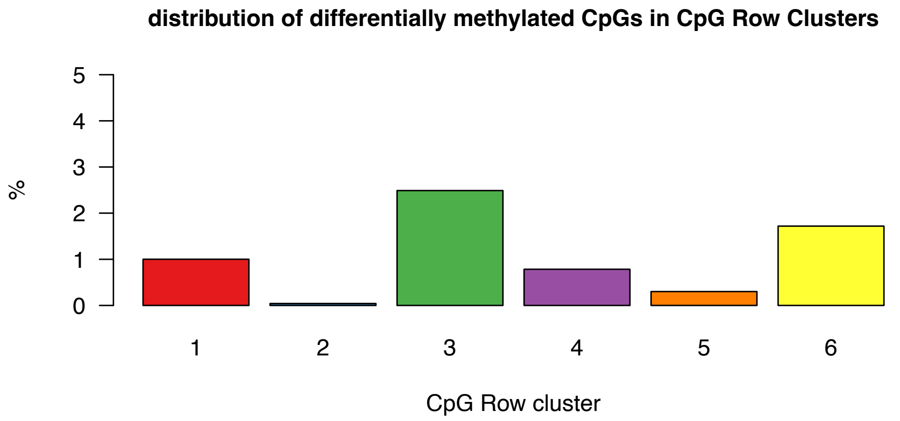

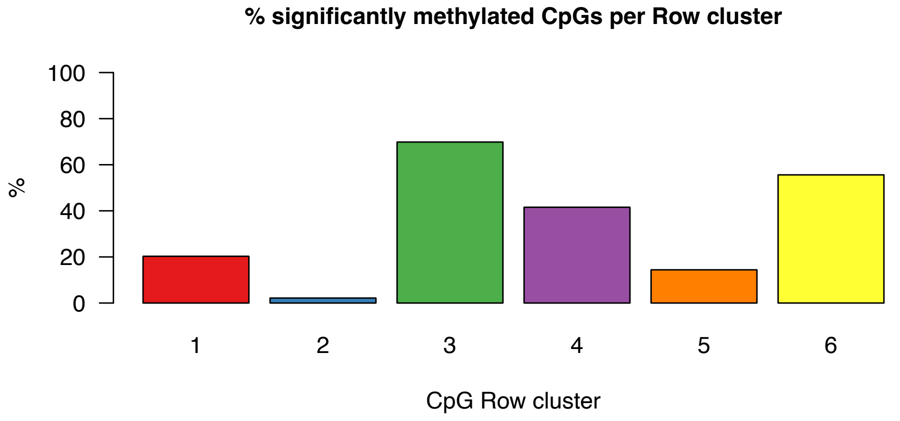

i)

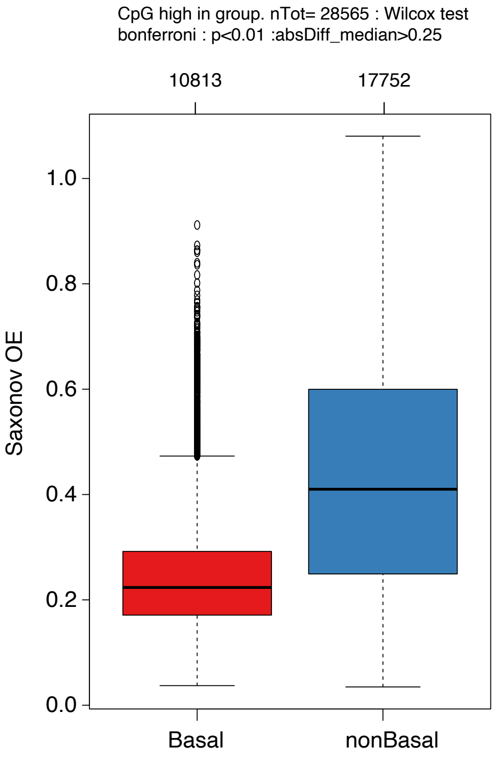

Supplementary Figure S4

j)

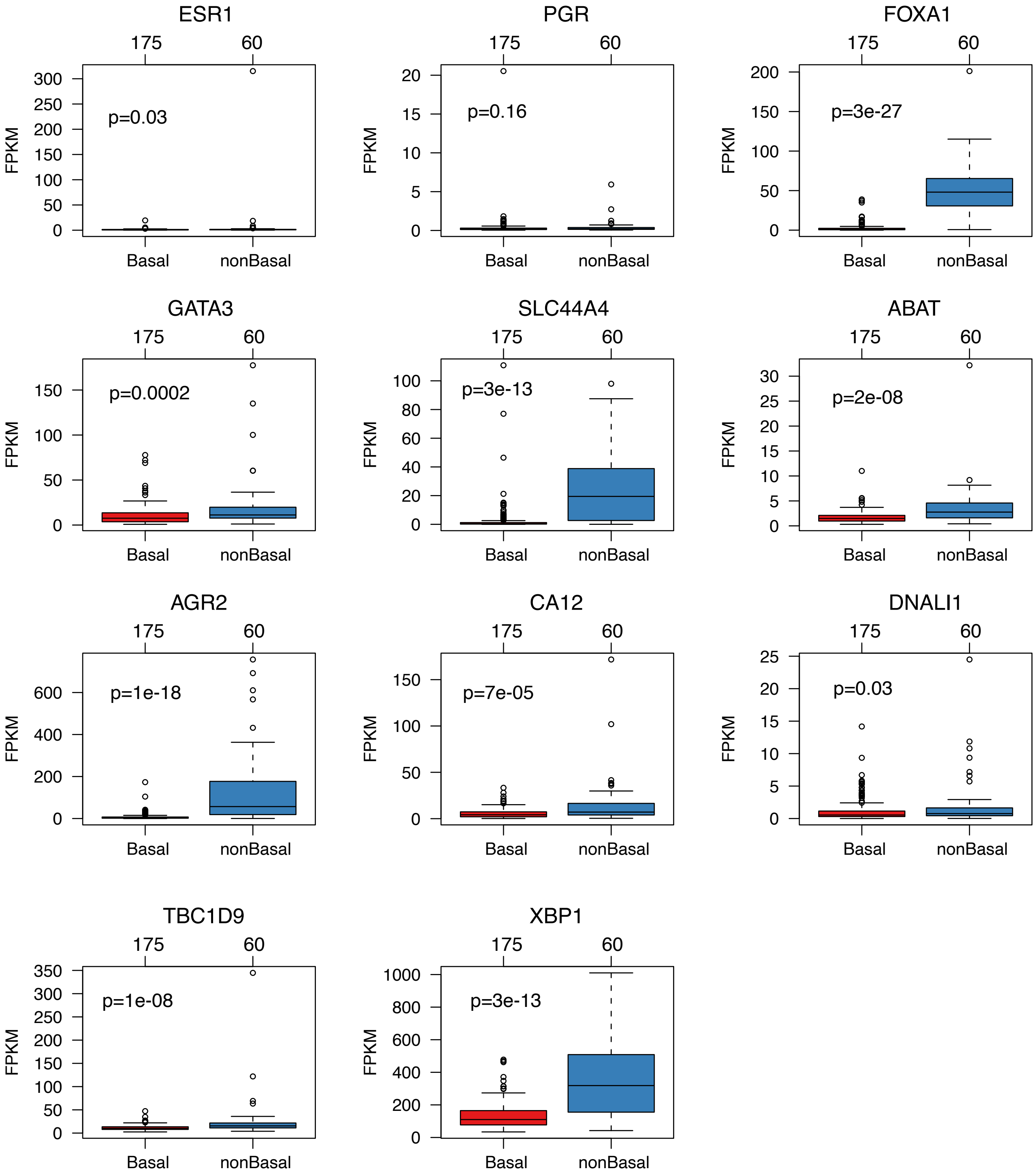

Supplementary Figure S4

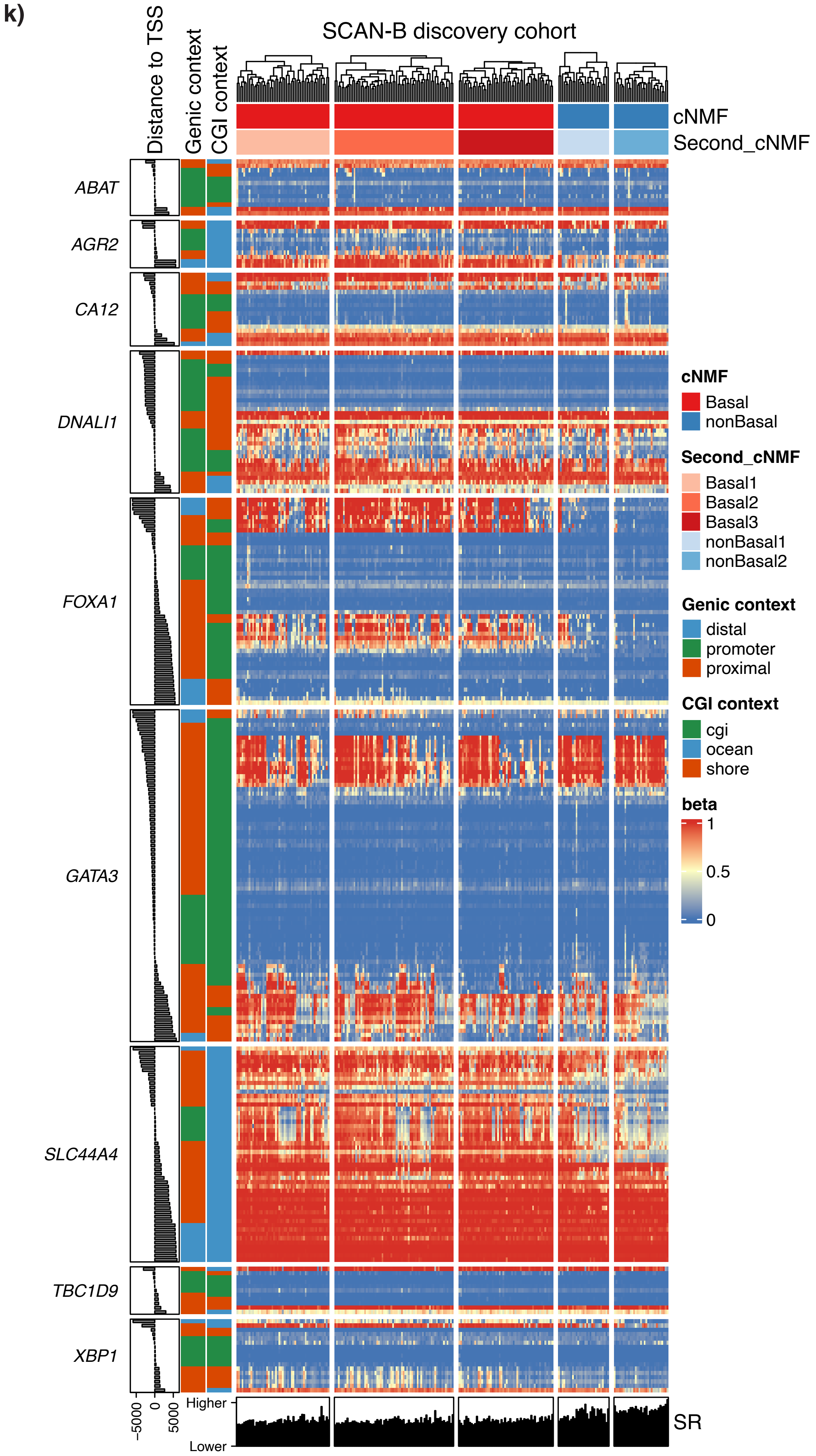

Supplementary Figure S4

I)

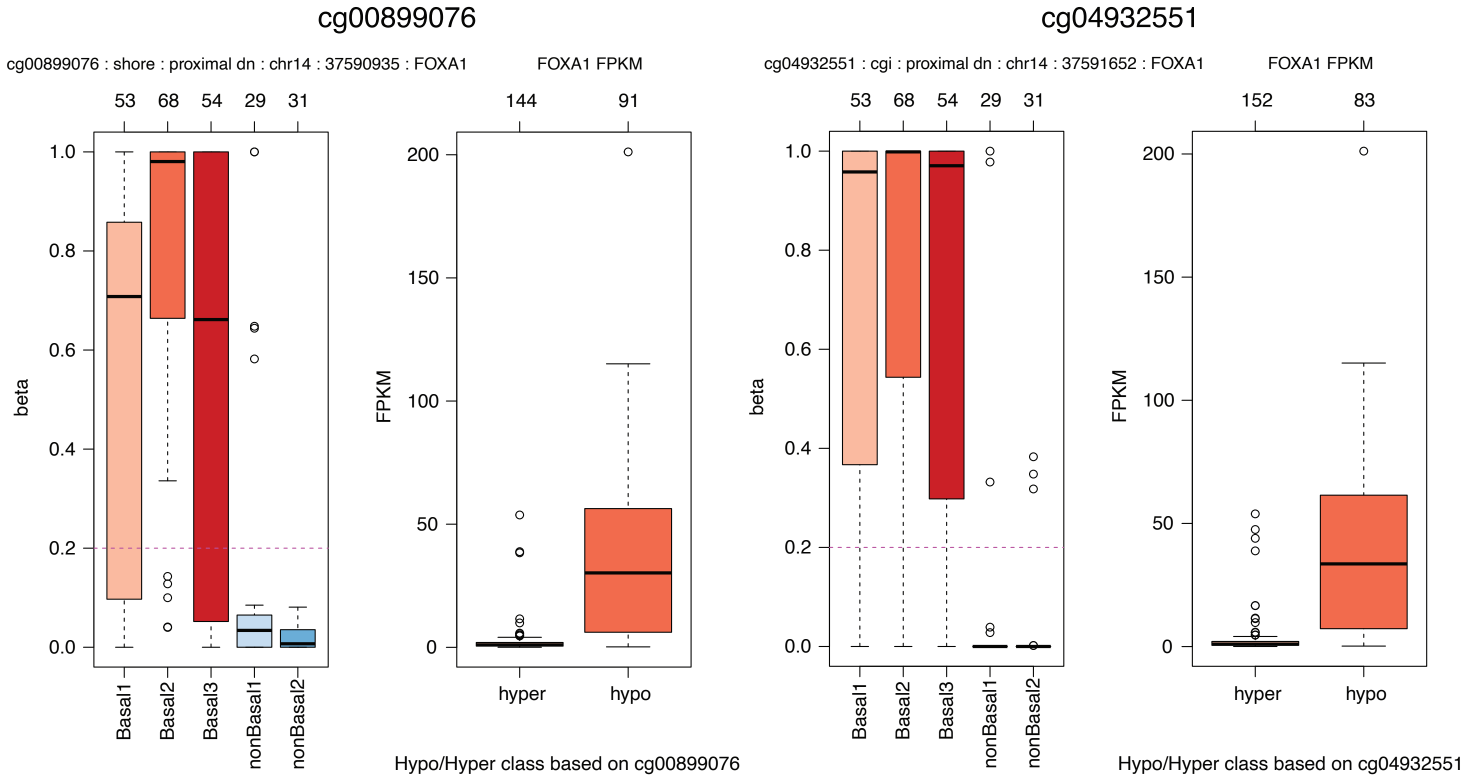

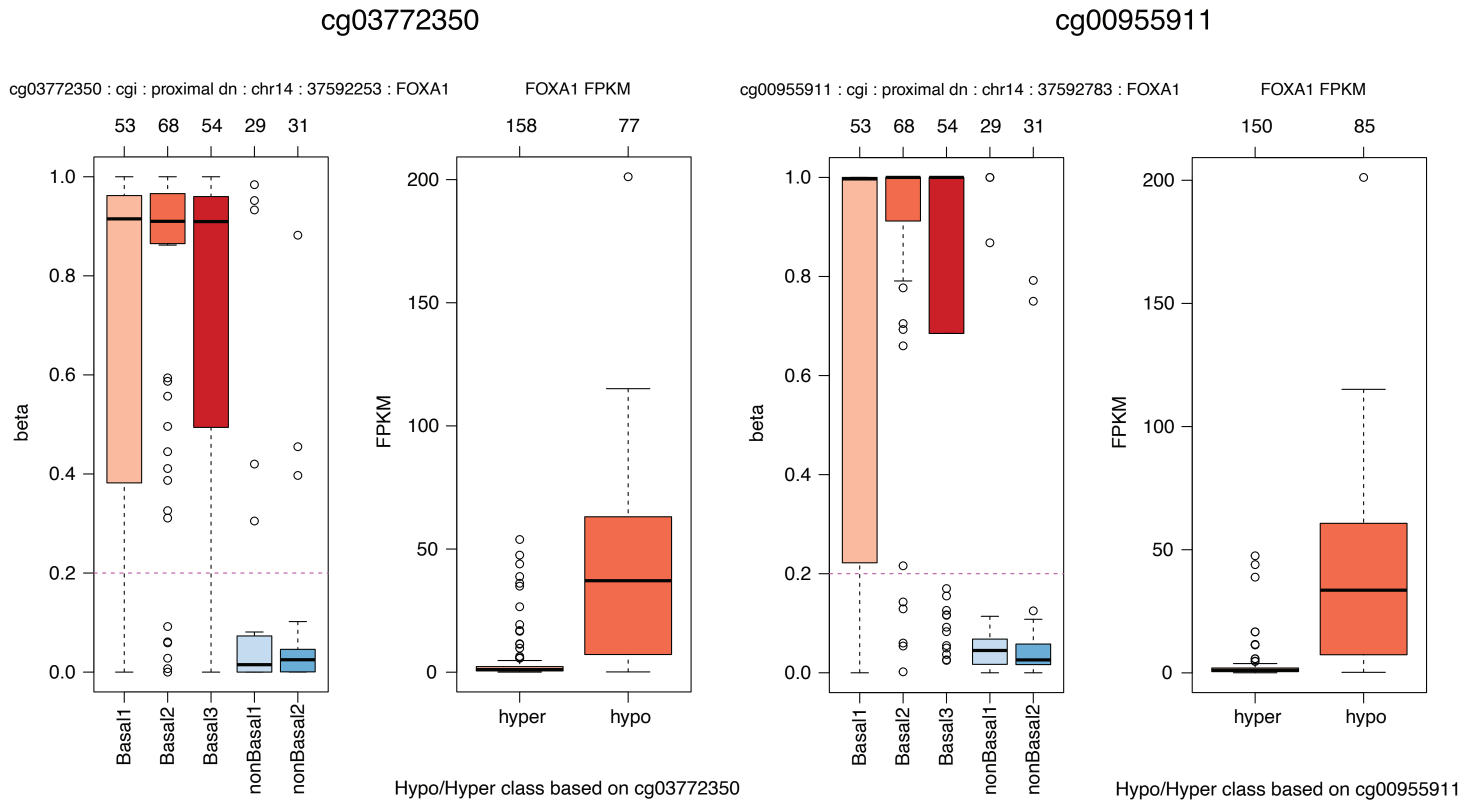

Supplementary Figure S4

m)

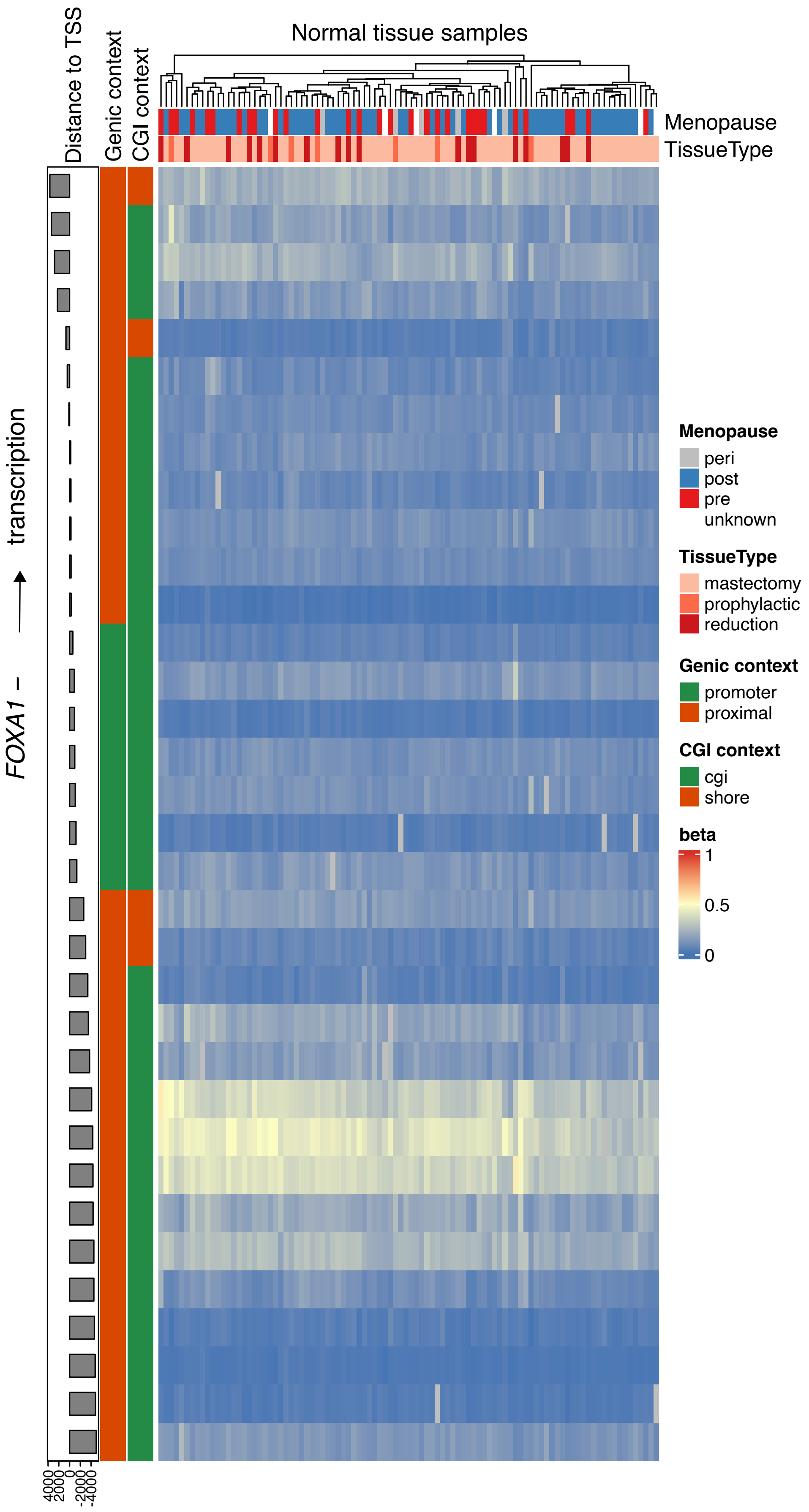

Supplementary Figure S4

n)

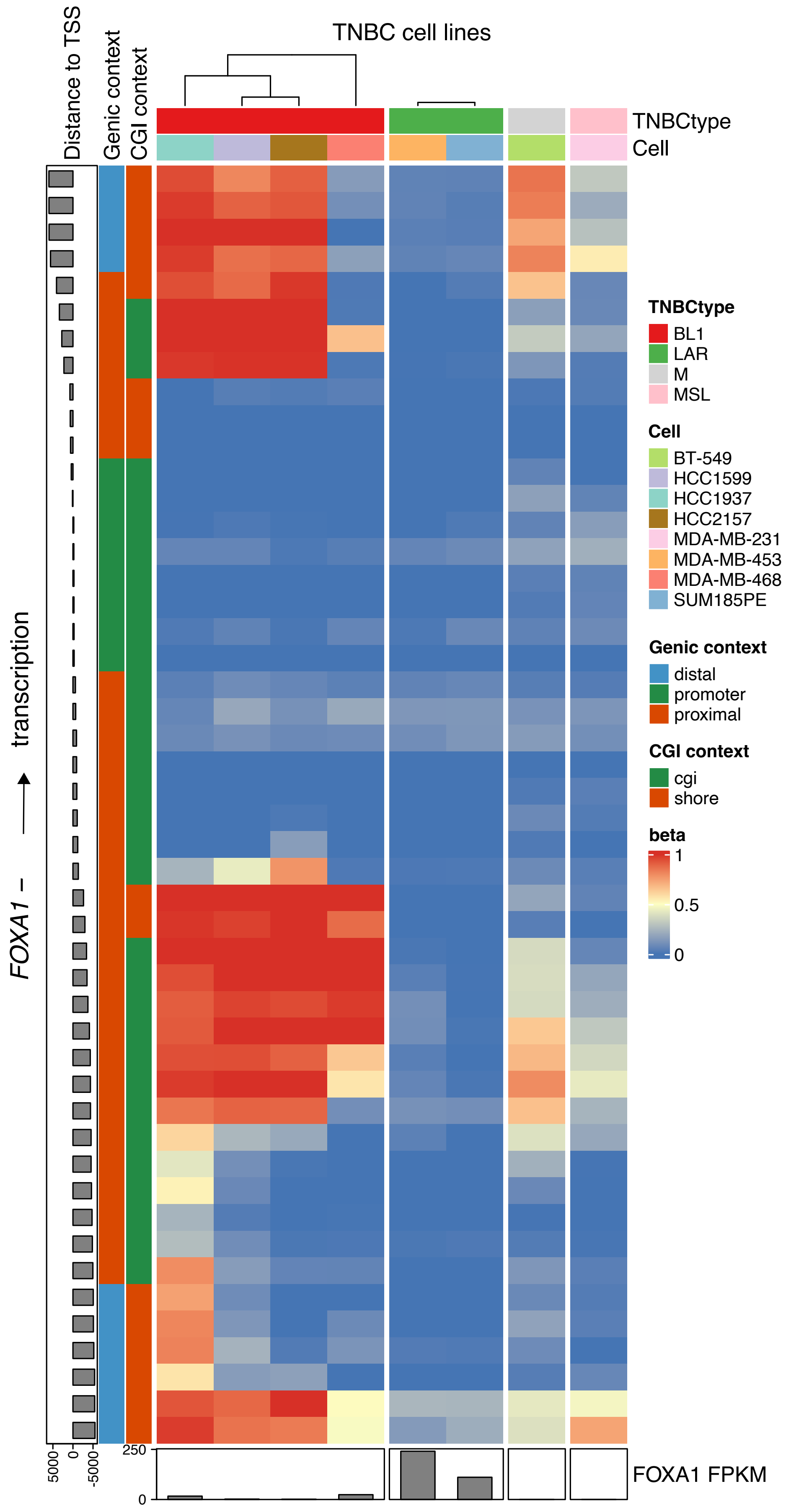

Supplementary Figure S4

o)

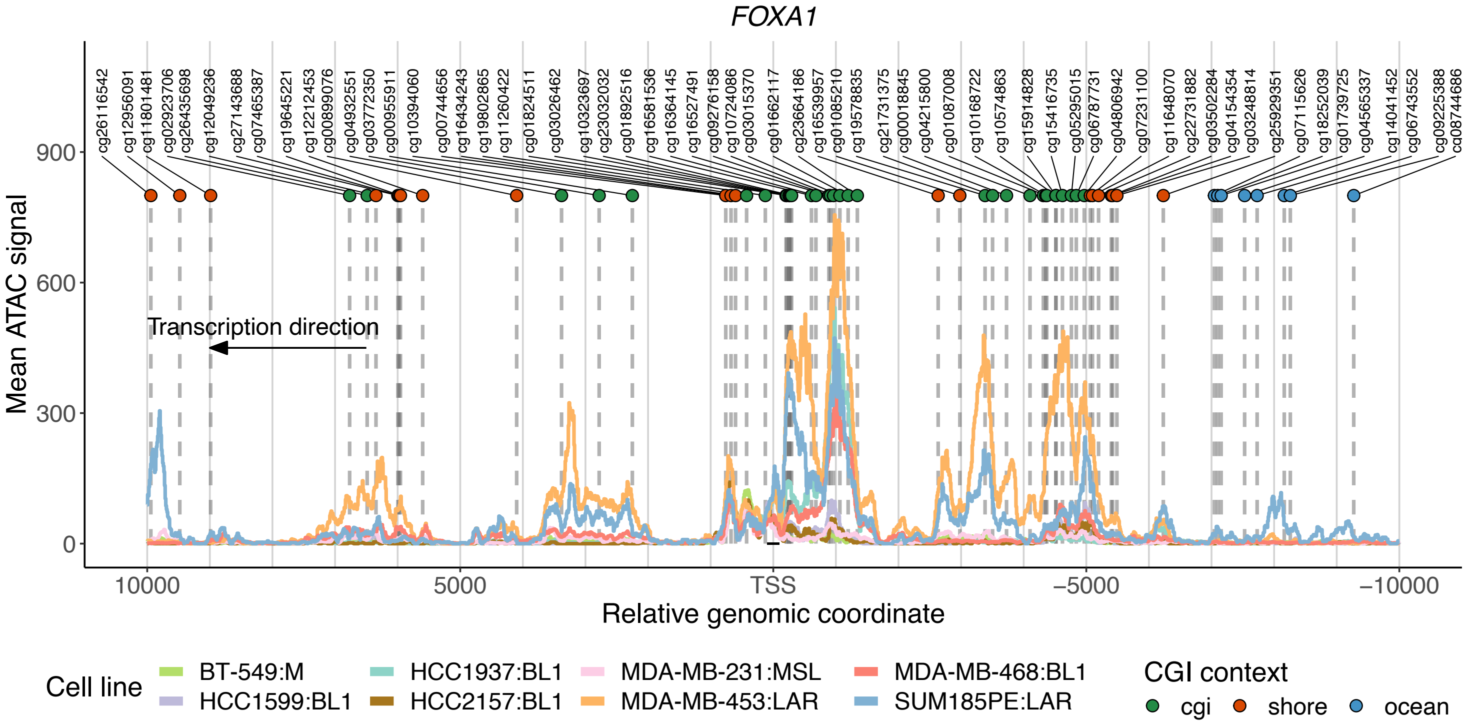

Supplementary Figure S4

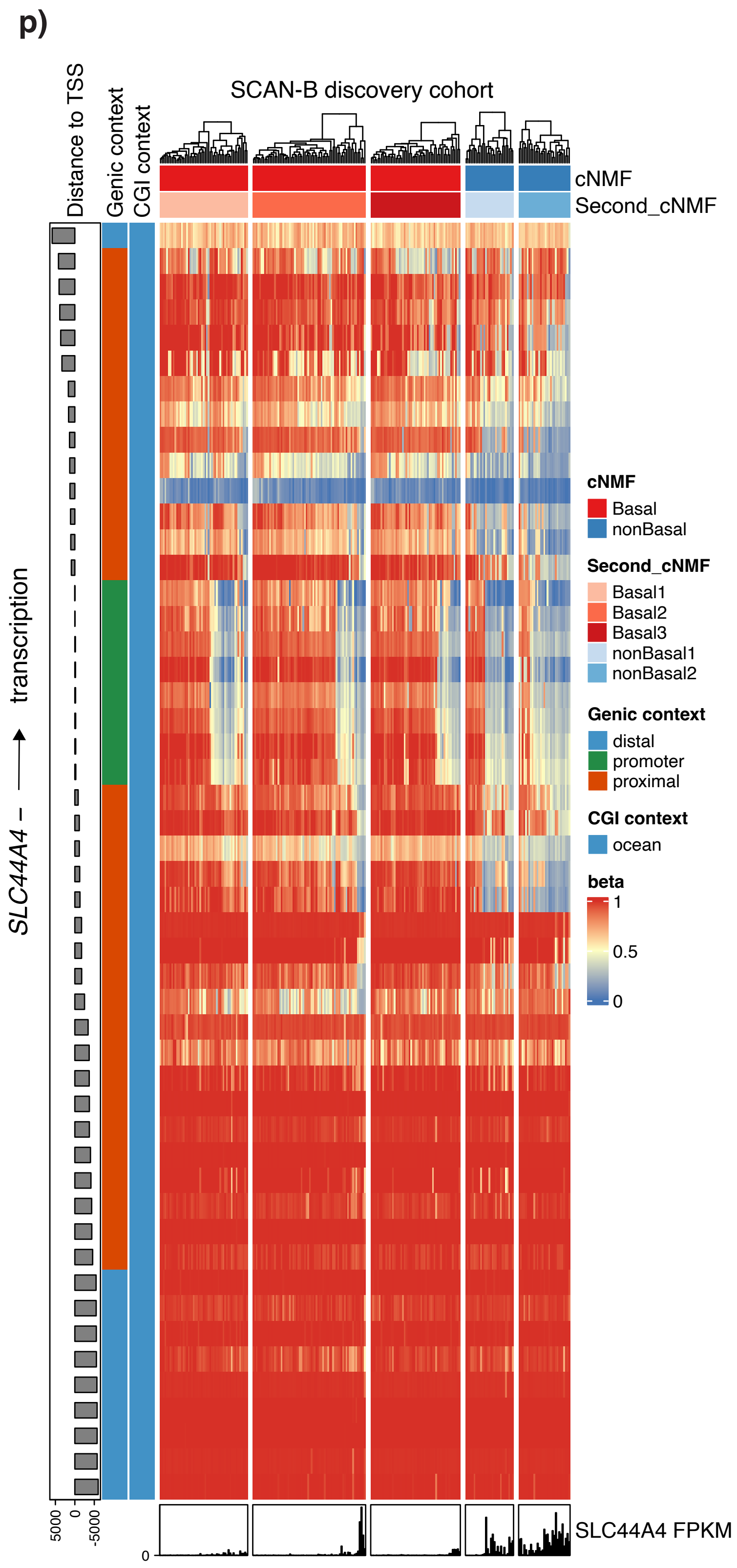

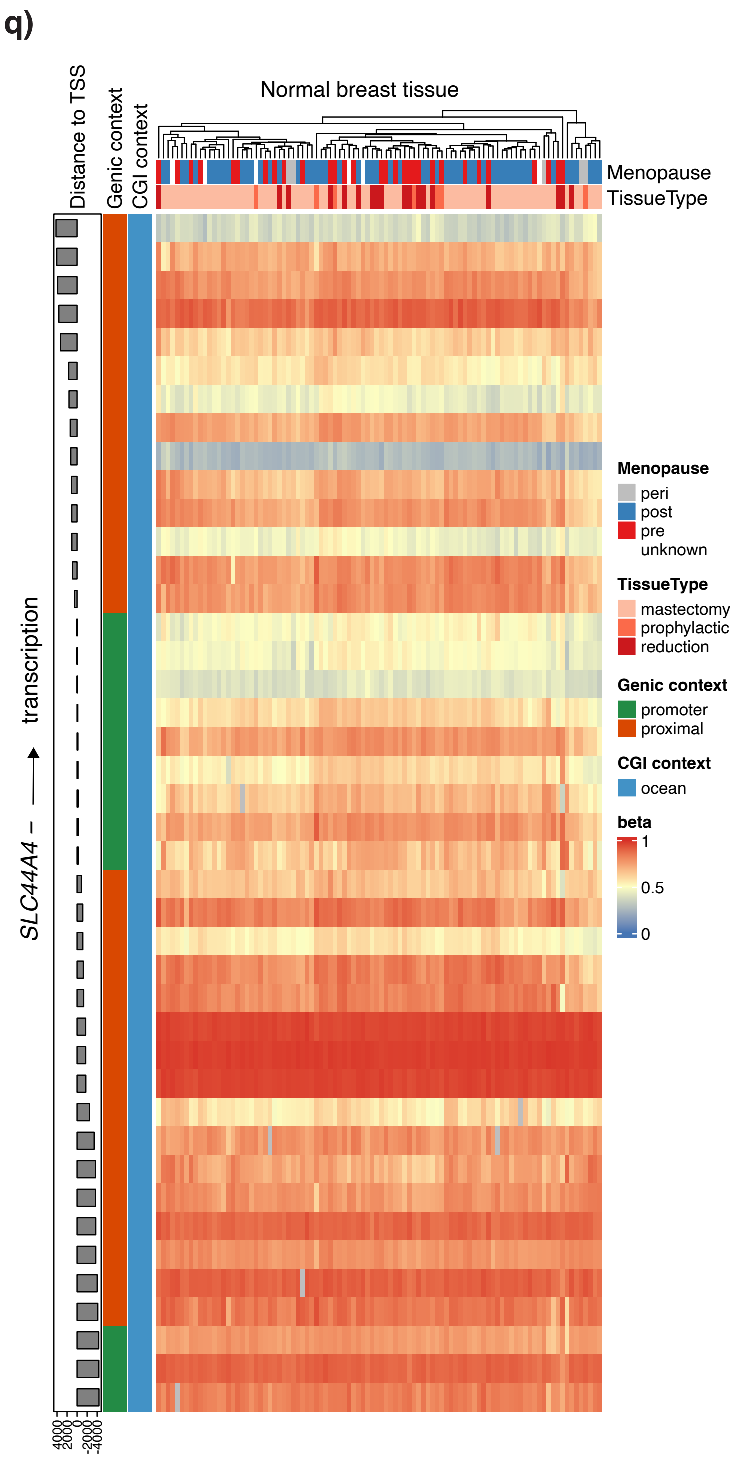

Supplementary Figure S4

r)

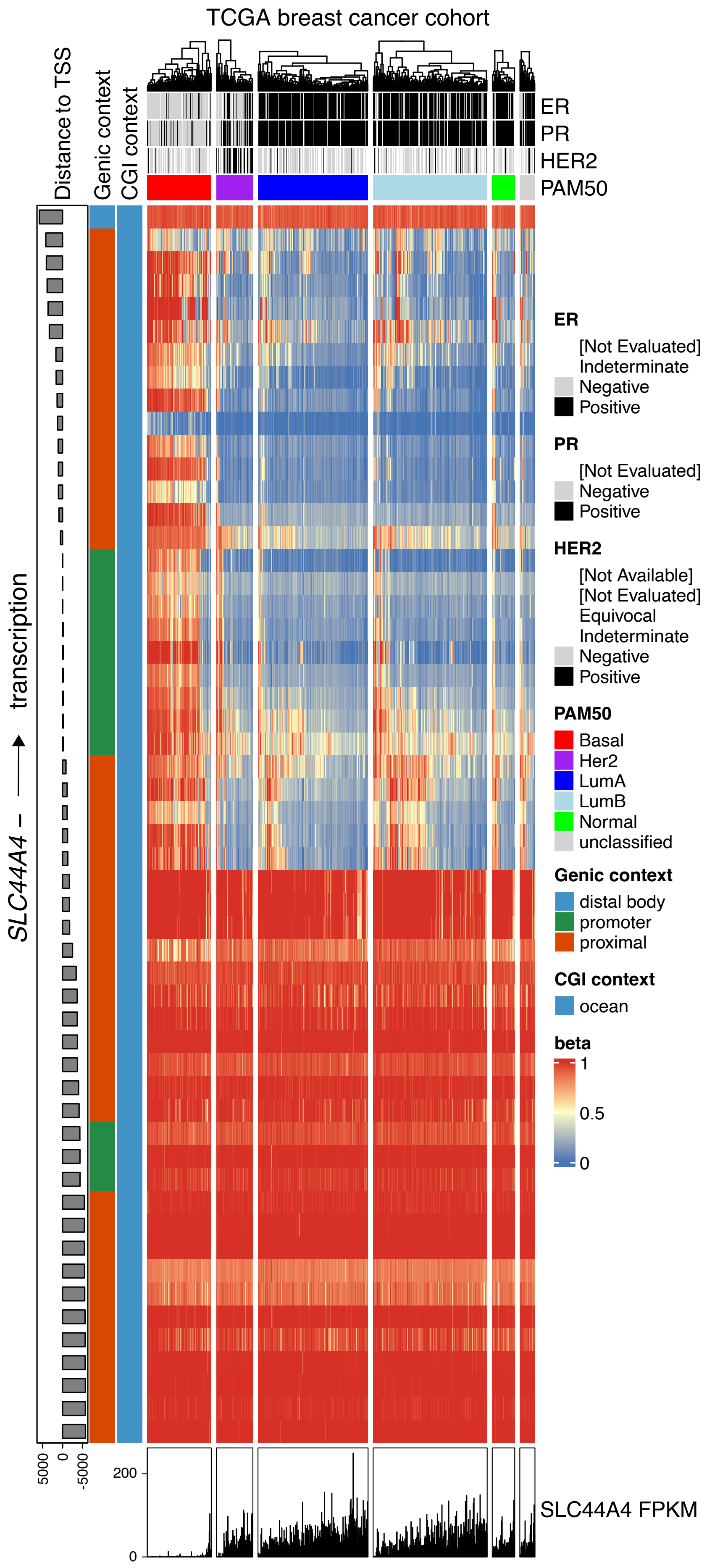

Supplementary Figure S4

s)

FOXC1

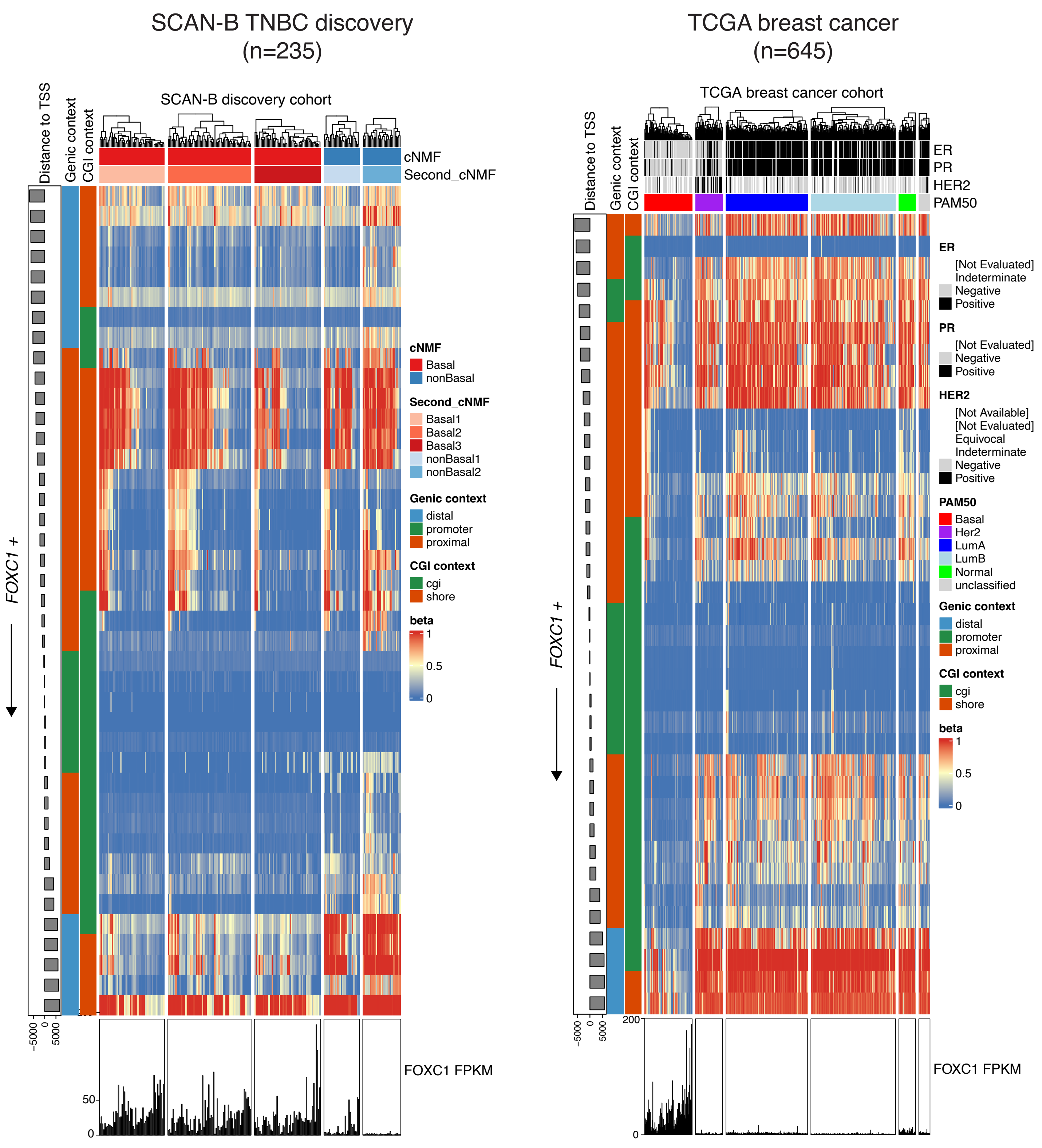

Supplementary Figure S4

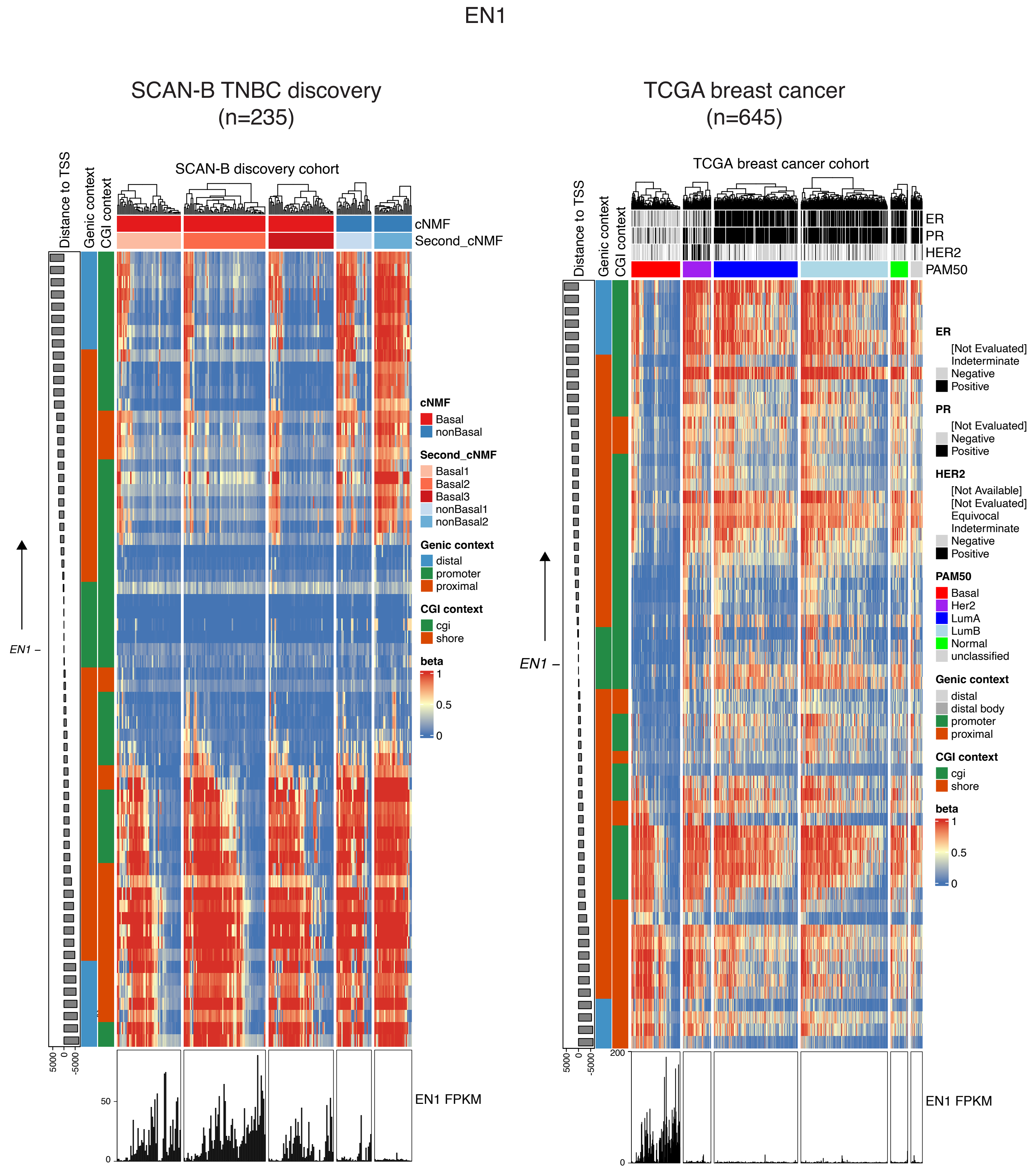

Supplementary Figure S4

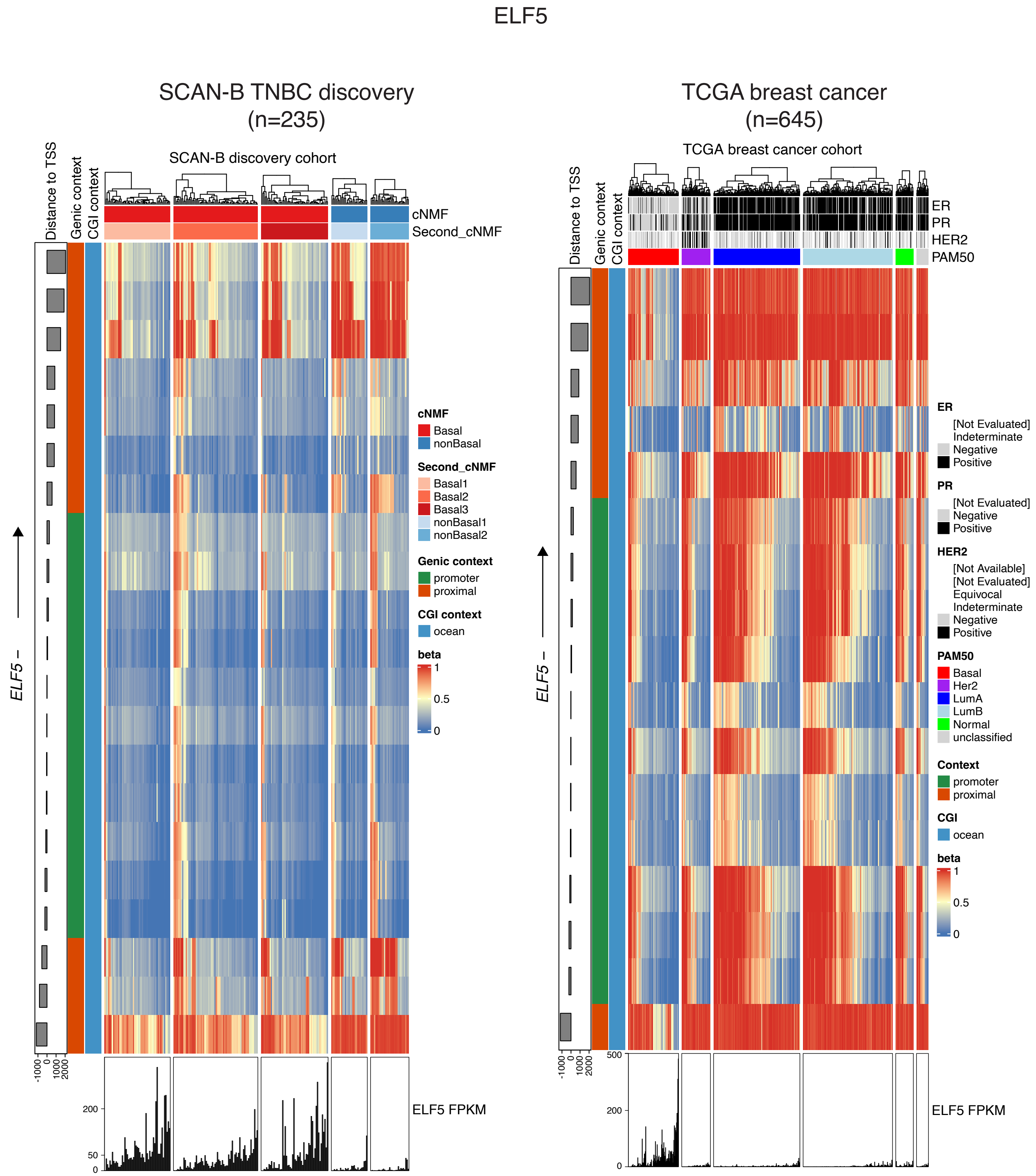

Supplementary Figure S4. Main Basal and nonBasal epitypes. (a) Classification agreement for NMF clustering of the 5000 most variant distal-ATAC CpGs across the 235 tumors using different numbers of clusters (k). **(b)** Consensus clusters for the two-group (k=2) NMF solution in (a) used to divide the 235-sample cohort into a Basal and a nonBasal epitype. **(c)** Composition of main Basal/nonBasal epitypes regarding PAM50 subtypes, TNBCtype subtypes, and HRD status. **(d)** *TP53* and PI3K pathway mutation (see Methods) percentages for main Basal/nonBasal epitypes. **(e)** Patient age at diagnosis for Basal/nonBasal epitypes. **(f)** Kaplan-Meier plot using distant relapse-free interval (DRFI) for chemotherapy-treated patients stratified by Basal (n=117 samples) and nonBasal (n=24 samples) epitypes. P-value calculated using the log-rank test. **(g)** Aggregated mean H3K27Ac signal across different non-malignant cell types for CpG positions in the different row clusters (i.e. summarizing and aggregating all CpGs in a row cluster) defined in main Figure 3a, showing that the signal is present in luminal progenitor cell lines, at varying strength, but not in lymphocytes like CD4 and CD8 cells. CpG coordinate 0 corresponds to the first CpG position. **(h)** Top panel: percentage of differentially methylated CpGs between Basal and nonBasal epitypes mapping to the 5000 most variant distal-ATAC CpGs per CpG cluster from Figure 3a. Bottom panel: percentage of CpGs in each CpG cluster from Figure 3a included in the set of differentially methylated CpGs between epitypes. **(i)** Saxonov observed/expected (OE) metric for CpGs differentially methylated between Basal and nonBasal tumors, stratified by whether the median group methylation was higher in Basal or nonBasal. **(j)** FPKM expression for *ESR1*, *PGR*, and 9 genes included in the steroid response metagene for the epitypes. Two-sided p-values calculated using Wilcoxon's test. **(k)** Promoter methylation maps of the 9 genes included in the steroid response metagene. Sample annotation tracks show the main Basal/nonBasal epitypes (cNMF) and their subgroups (Second_cNMF, Basal1-3 and nonBasal1-2). CpGs for each gene are ordered according to distance from the transcription start site (TSS) and are annotated for CpG contexts. Bottom panel shows the corresponding steroid response (SR) metagene scores for each tumor. **(l)** Selected plots of beta values and FPKM expression for 4 CpGs in *FOXA1* labelled in main Figure 3f for the main epitypes. Right panels show mRNA expression versus an inferred hypo/hypermethylation status using beta=0.2 as cutoff (dotted line on left panels). **(m)** Promoter methylation for *FOXA1* in 96 normal breast specimens. CpGs are ordered in increasing base pair position. *FOXA1* strand direction is minus (-). **(n)** Promoter methylation for *FOXA1* in 8 TNBC cell lines. CpGs are ordered in increasing base pair position. *FOXA1* strand direction is minus (-). **(o)** Mean ATAC-seq signal along the *FOXA1* promoter region for the 8 TNBC cell lines. CpG positions and CGI contexts are superimposed. TSS = transcription start site. **(p)** Promoter methylation for *SLC44A4* in 235 SCAN-B tumors. CpGs ordered according to increasing base pairs. *SLC44A4* strand direction is minus (-). Bottom panel shows *SLC44A4* FPKM expression. **(q)** Promoter methylation for *SLC44A4* in 96 normal breast specimens. CpGs ordered according to increasing base pairs. *SLC44A4* strand direction is minus (-). **(r)** Promoter methylation for *SLC44A4* in 645 TCGA tumors. CpGs ordered according to increasing base pairs. *SLC44A4* strand direction is minus (-). Bottom panel shows corresponding *SLC44A4* FPKM expression. **(s)** Promoter methylation for *FOXC1, EN1,* and *ELF5* in 235 SCAN-B tumors and 645 TCGA tumors. CpGs ordered according to increasing base pairs in each plot. Arrows indicate strand direction.

All reported p-values from statistical tests are two-sided if not otherwise specified. Boxplot elements correspond to: (i) center line = median, (ii) box limits = upper and lower quartiles, (iii) whiskers = 1.5x interquartile range. Top-axis in boxplots reports group sizes.

Supplementary Figure S5

a)

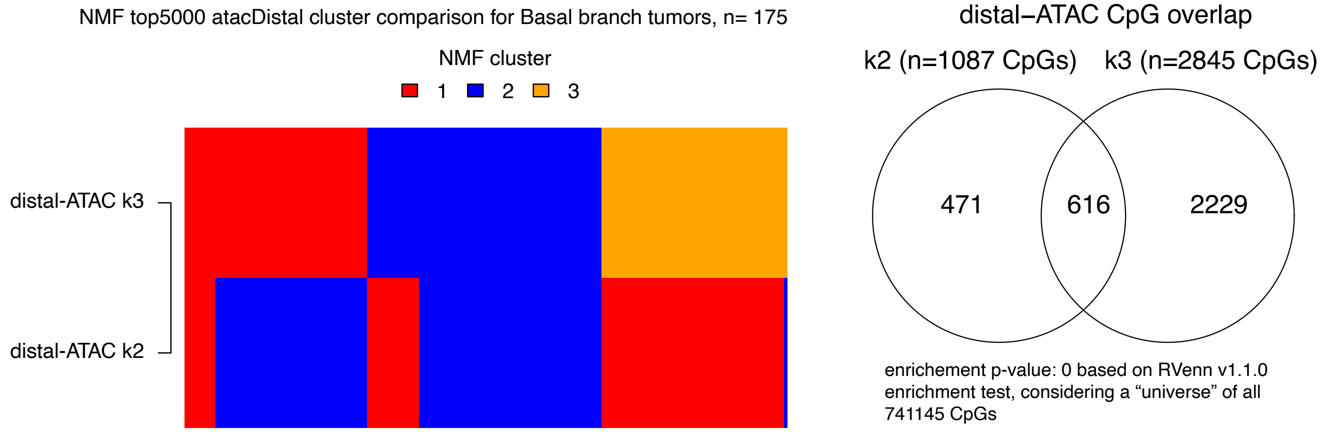

b)

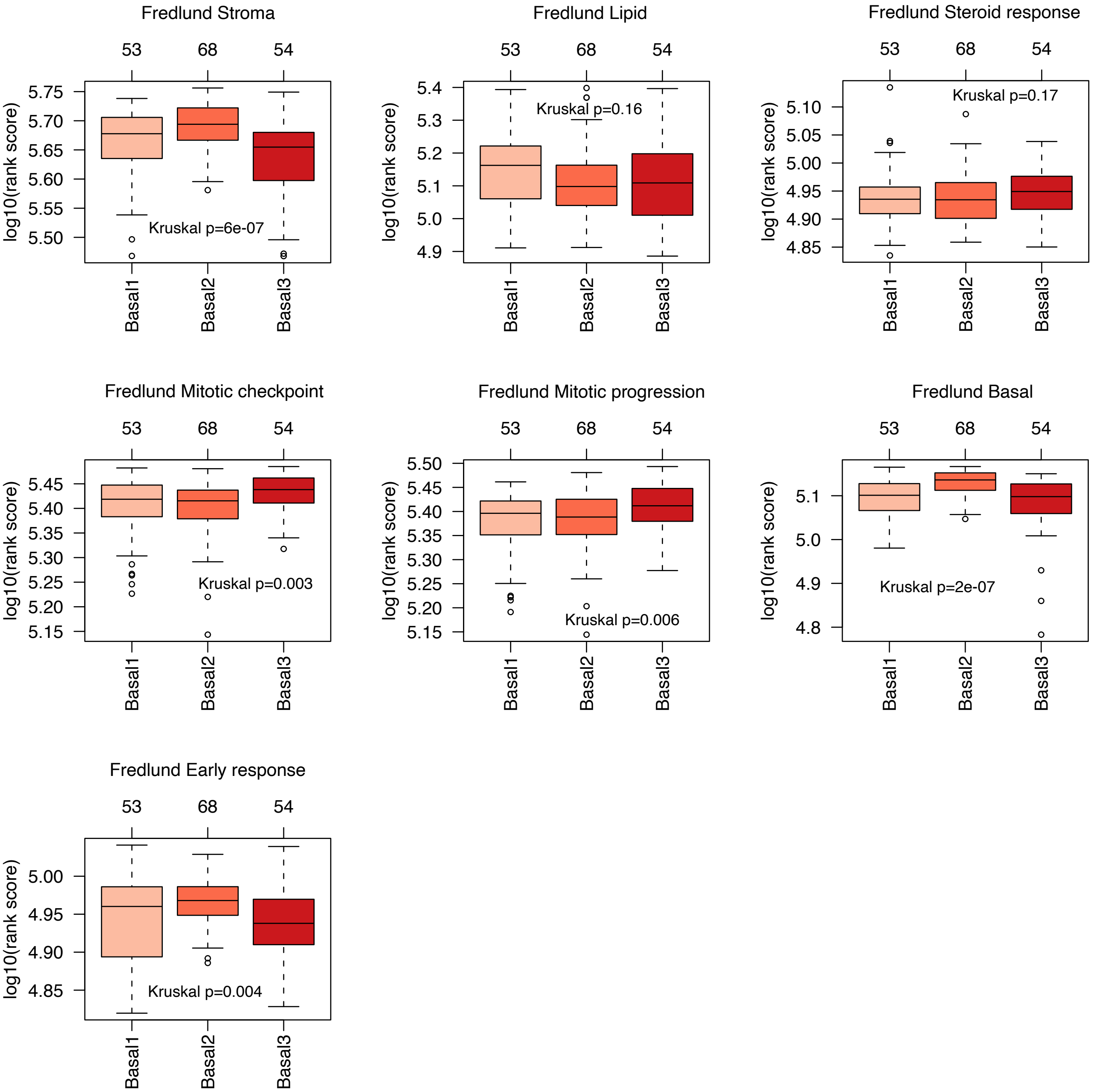

Supplementary Figure S5

c)

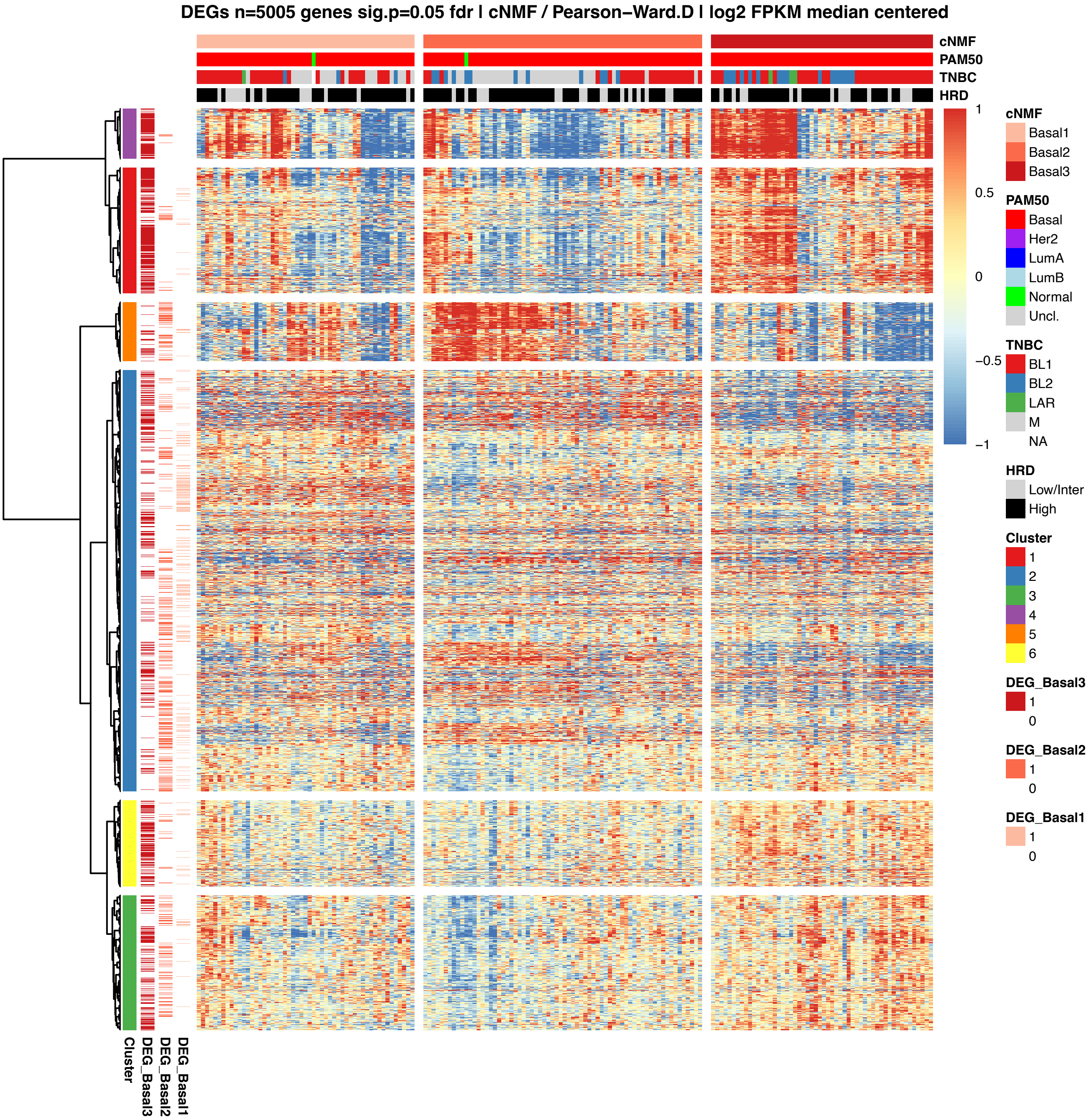

Supplementary Figure S5

d)

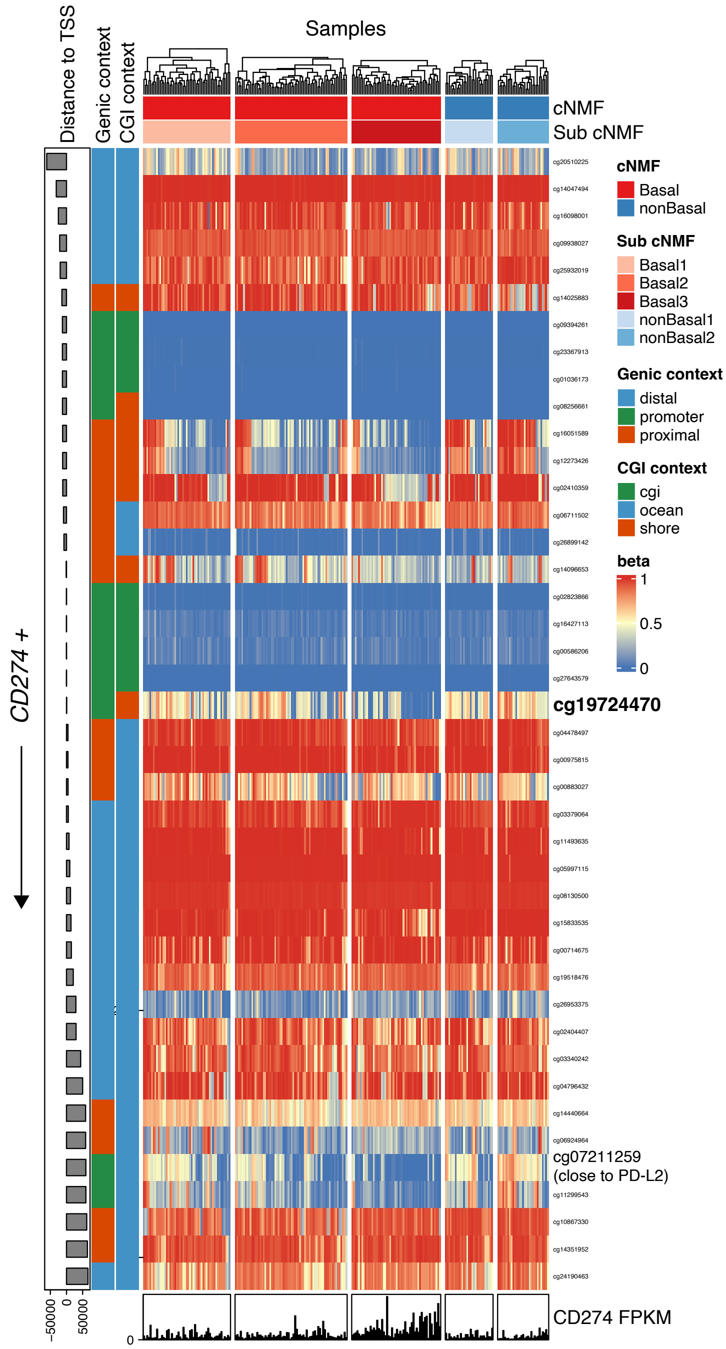

e)

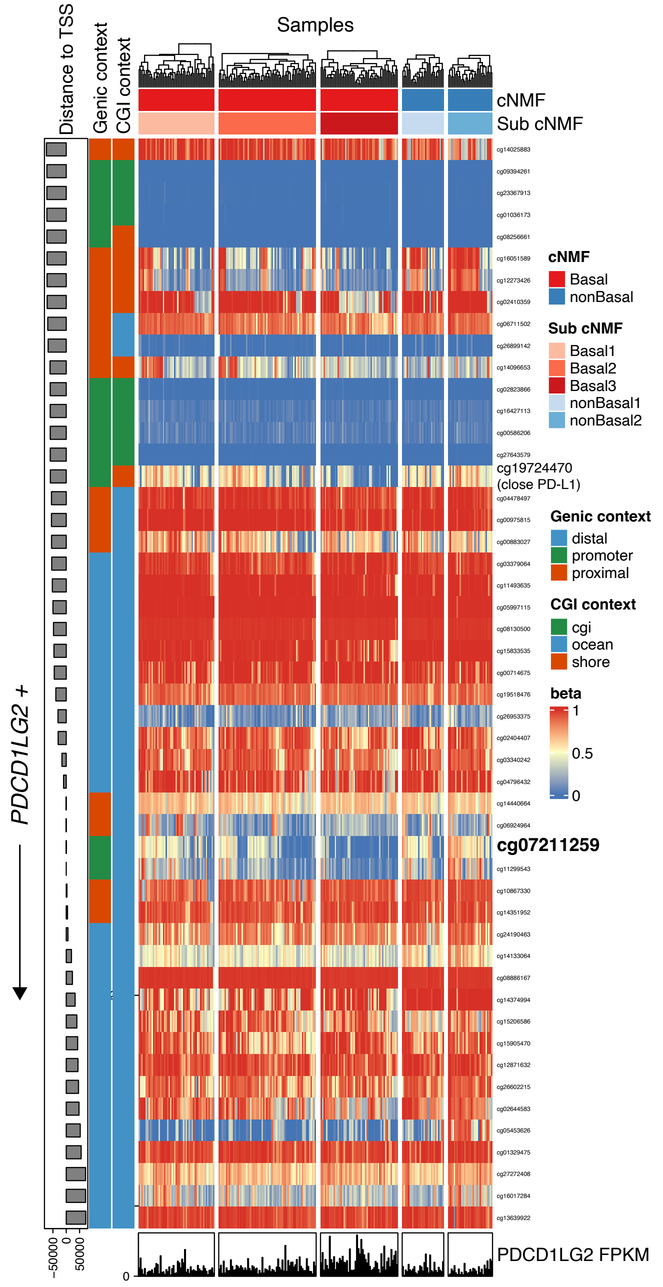

Supplementary Figure S5

f)

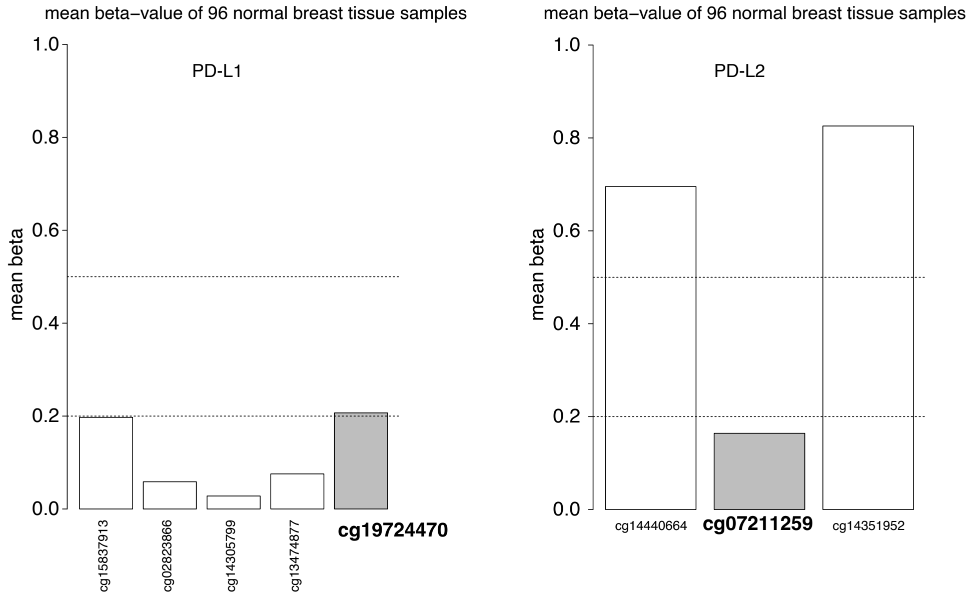

g)

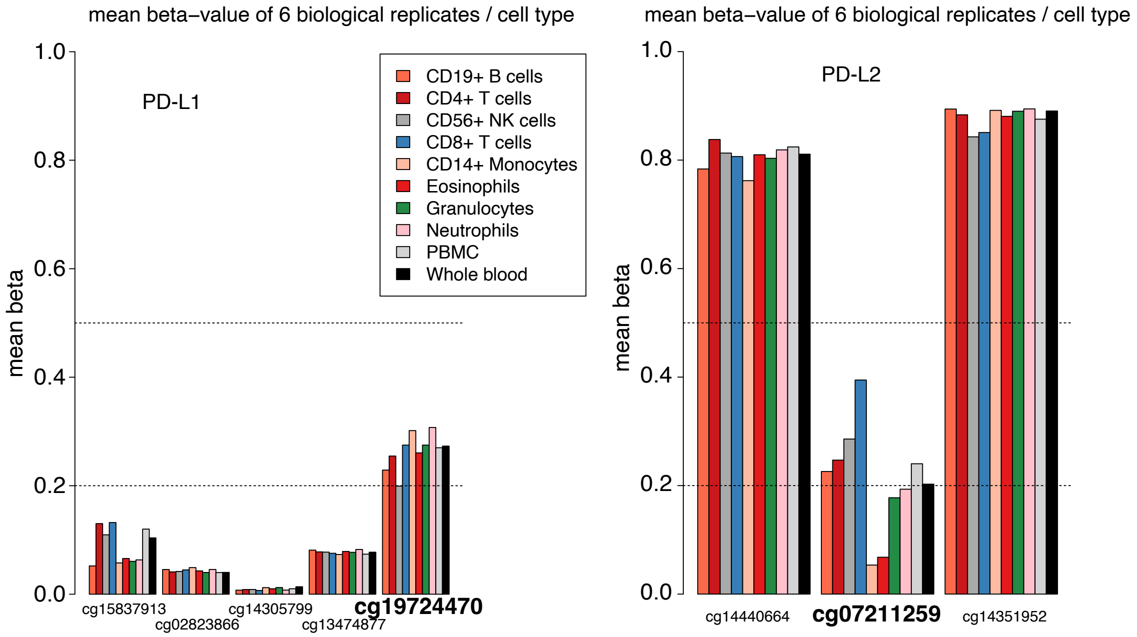

h)

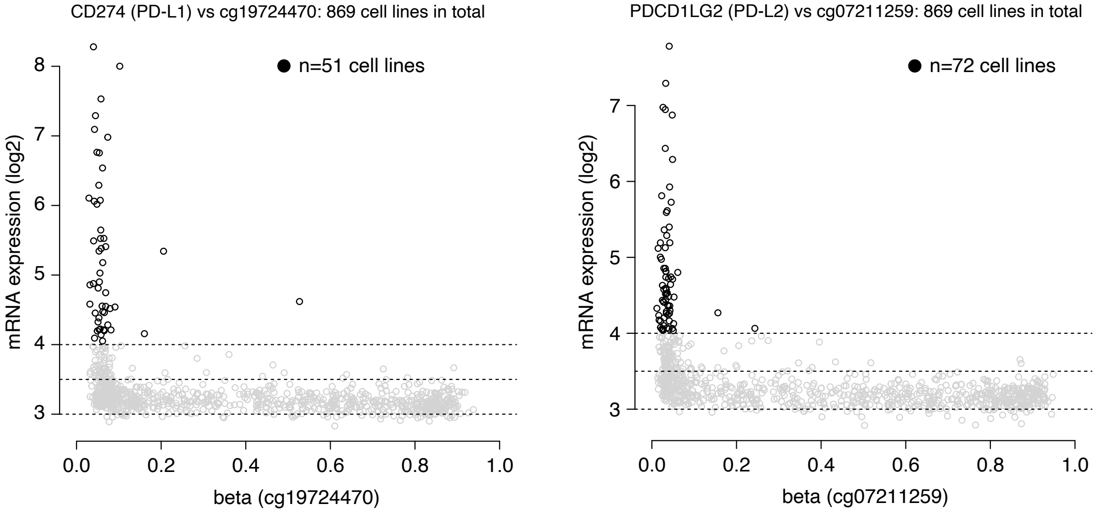

Supplementary Figure S5

i)

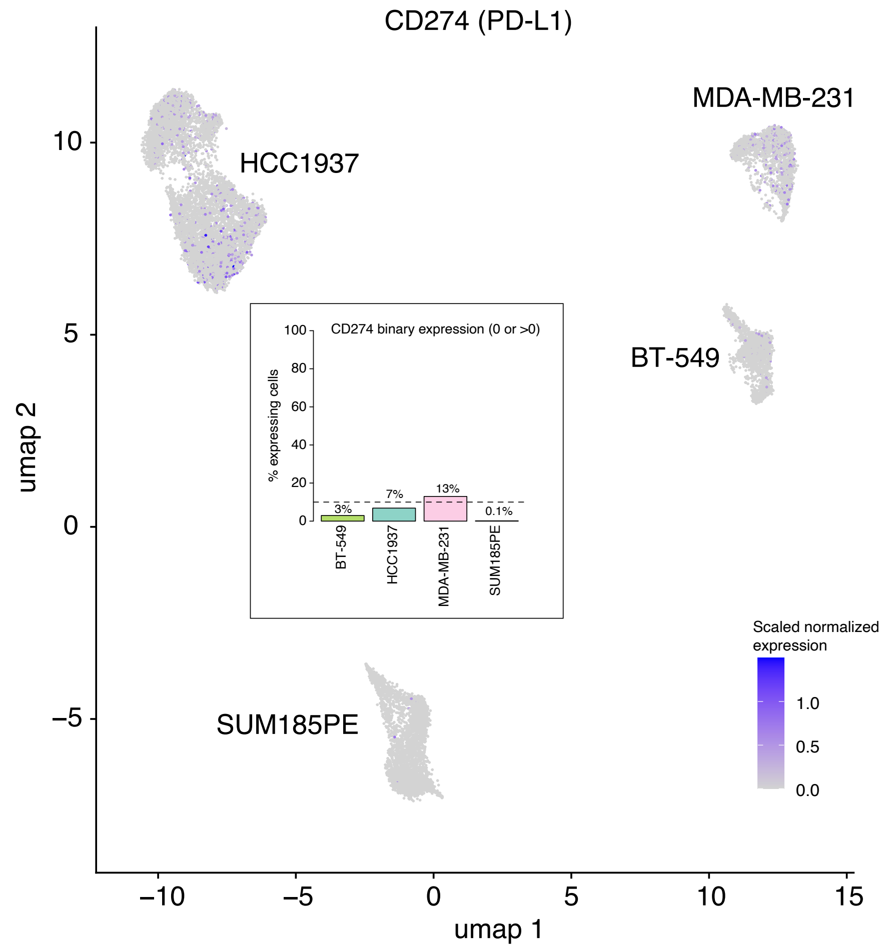

j)

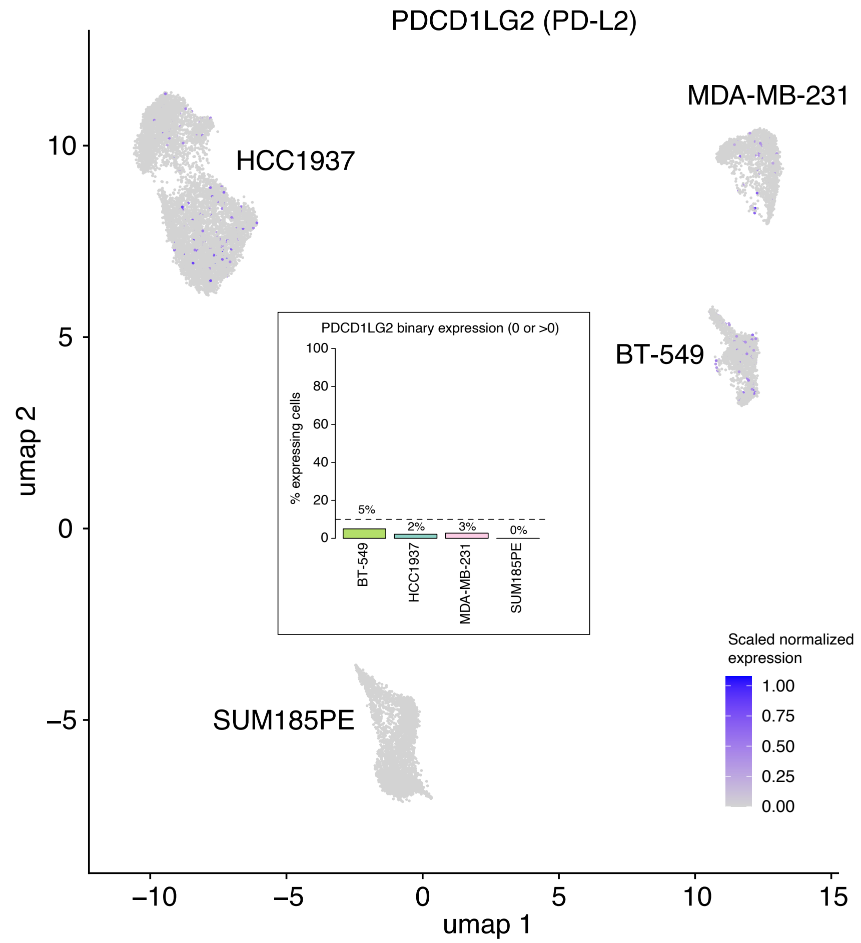

Supplementary Figure S5. Subgroups within the Basal epitype. (a) Left: comparison of NMF classifications using different cluster numbers (k) and 5000 distal-ATAC CpGs of 175 tumors in the main Basal epitype. Right: Venn diagram showing partial overlap of differentially methylated CpGs between the k=2 and k=3 NMF solutions. **(b)** Boxplots of 7 metagene scores for the Basal subgroups. Two-sided p-values computed using Kruskal-Wallis test. **(c)** Heatmap of differentially expressed genes (FDR-adjusted Kruskal-Wallis test, $p<0.05$, >1 FPKM difference) between Basal1-3 tumors. Row annotations indicate to which subgroup a differentially expressed gene (DEG) is connected. **(d-e)** Promoter methylation for (d) *CD274* (*PD-L1*) and (e) *PDCD1LG2* (*PD-L2*). Two CpGs mentioned in main text are highlighted. Strand direction is plus (+). CpGs are ordered according to ascending genomic position. **(f)** Mean beta value for CpGs in promoter regions of *PD-L1* and *PD-L2* across 96 normal breast tissue specimens. CpGs described in main text are highlighted. **(g)** Mean beta value for different immune and blood cell types for CpGs in promoter regions of *PD-L1* and *PD-L2* . CpGs described in main text are highlighted. For each immune cell type, the mean value is based on 6 biological replicates. **(h)** Beta values and mRNA expression for CpGs associated with PD-L1 and PD-L2 in 869 cancer cell lines of several malignancies. **(i)** UMAP plot of single cell RNAseq (scRNAseq) data for four TNBC cell lines, showing normalized and scaled expression of *CD274* (*PD-L1*). Inserted bar plot shows the proportion of cells with a binary expression of the gene (0 expression or >0) per cell line. **(j)** Same plot as in (i) but for *PDCD1LG2* (*PD-L2*).

All reported p-values from statistical tests are two-sided if not otherwise specified. Boxplot elements correspond to: (i) center line = median, (ii) box limits = upper and lower quartiles, (iii) whiskers = 1.5x interquartile range. Top-axis in boxplots reports group sizes.

Supplementary Figure S6

a)

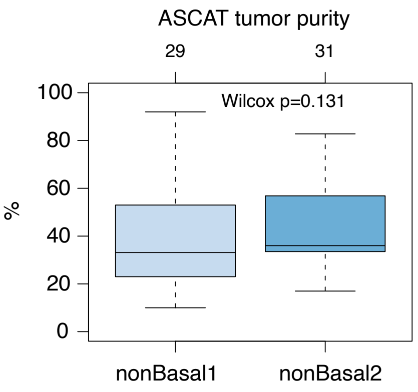

b)

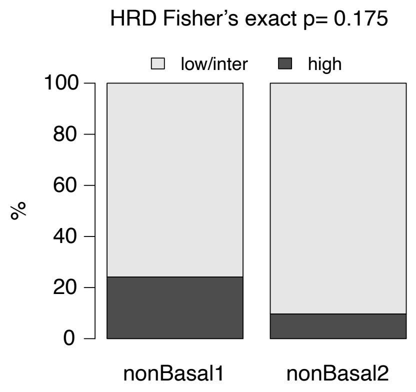

c)

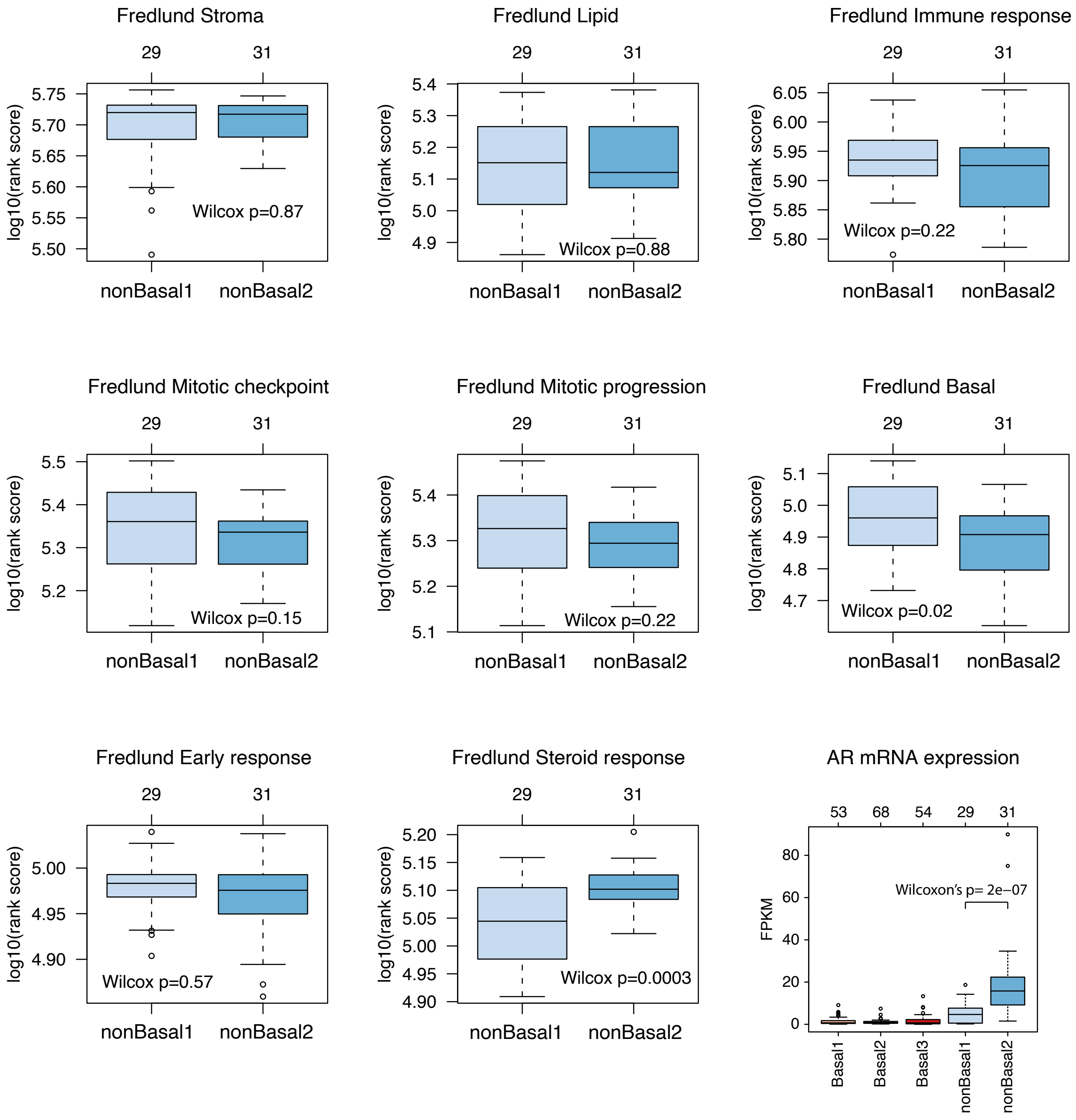

Supplementary Figure S6

d)

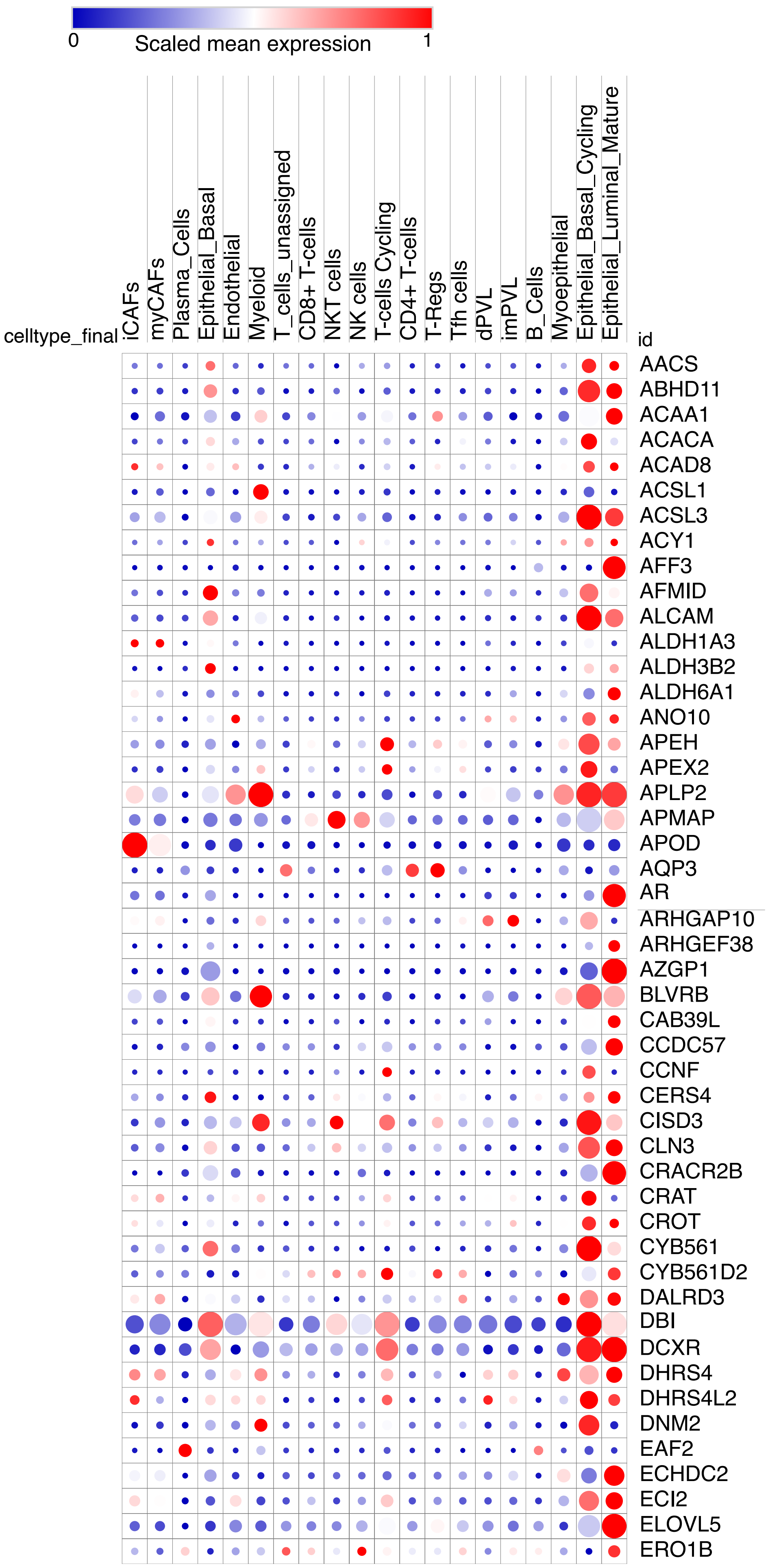

Supplementary Figure S6

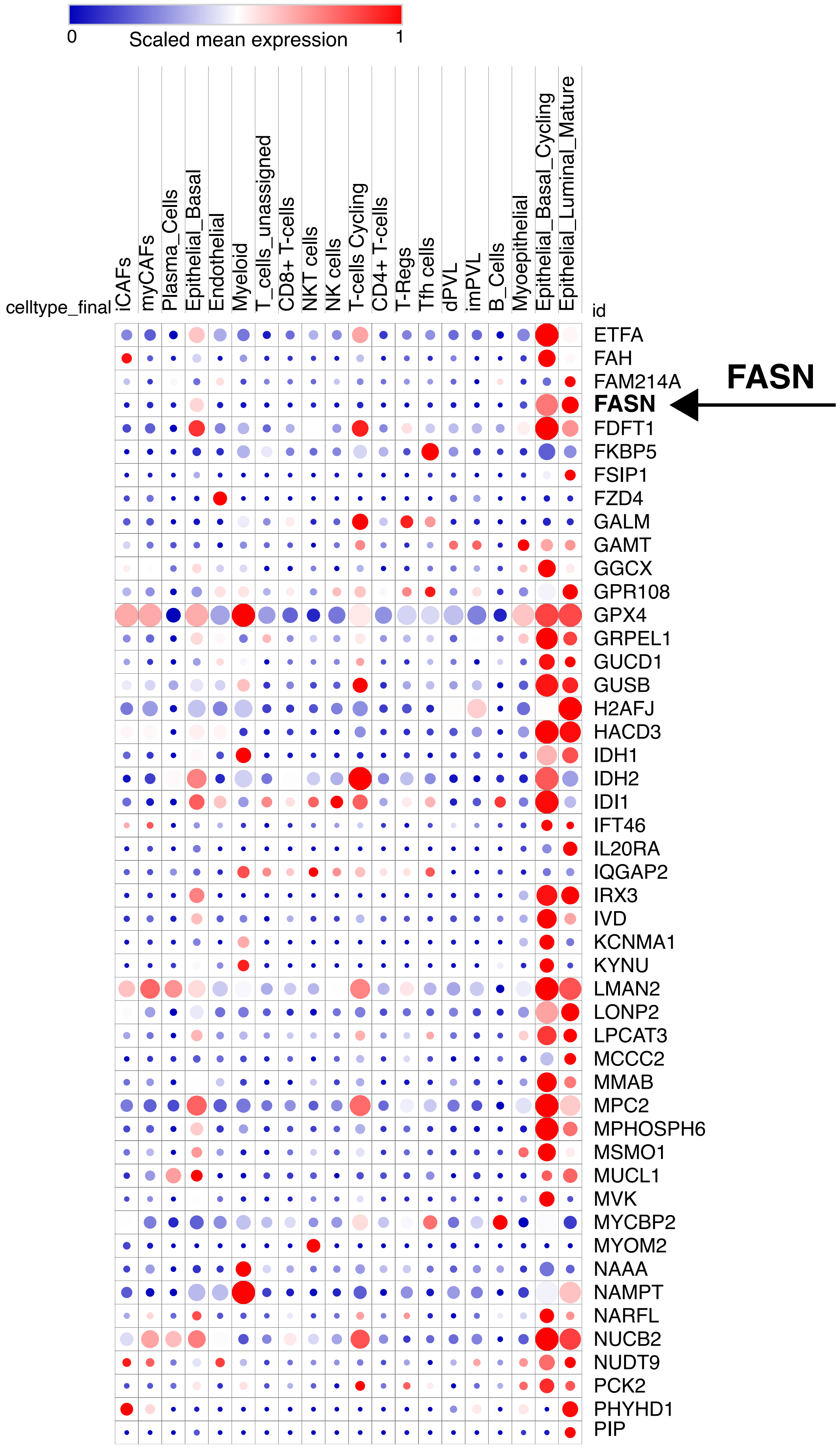

Supplementary Figure S6

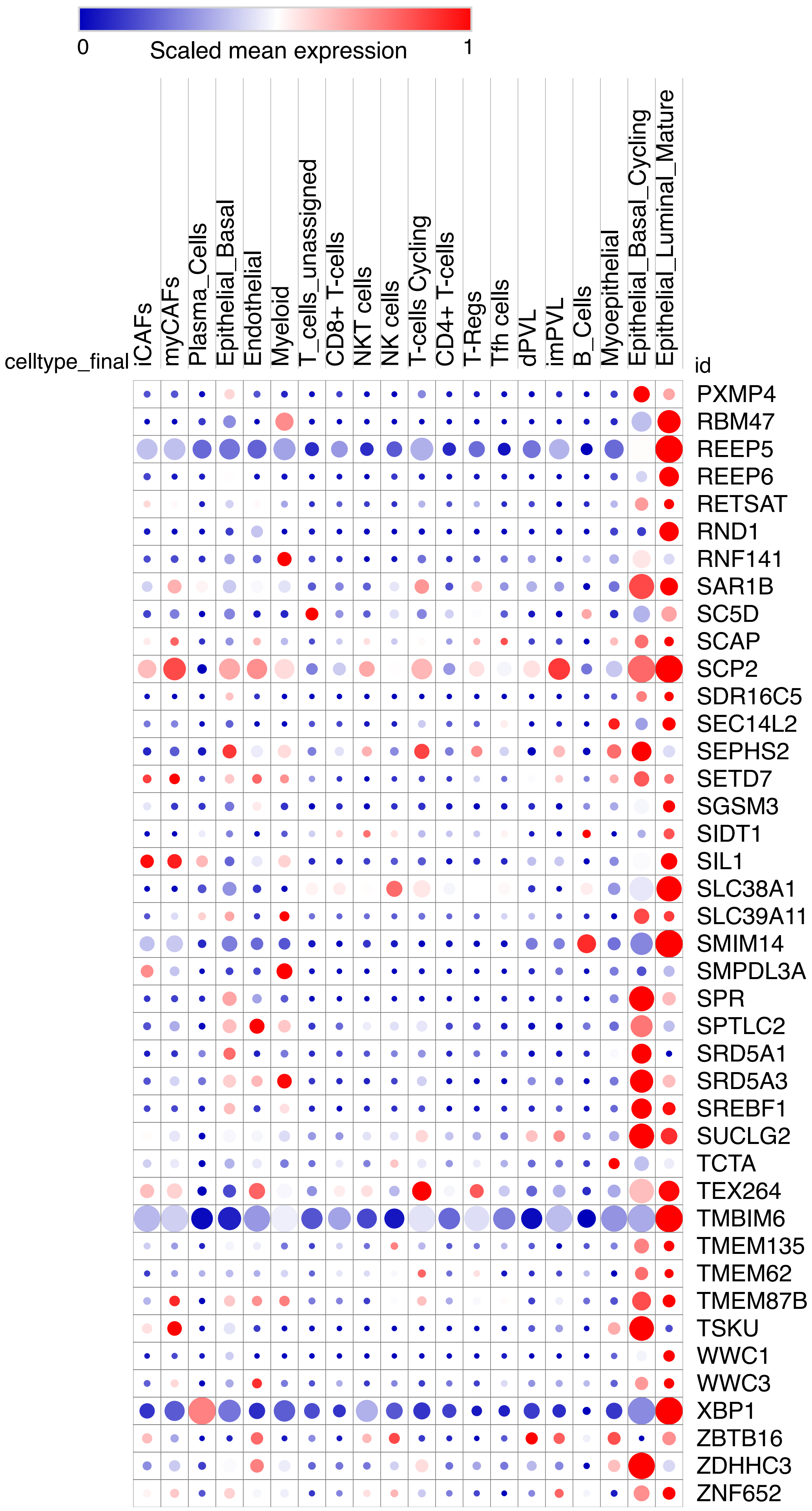

Supplementary Figure S6

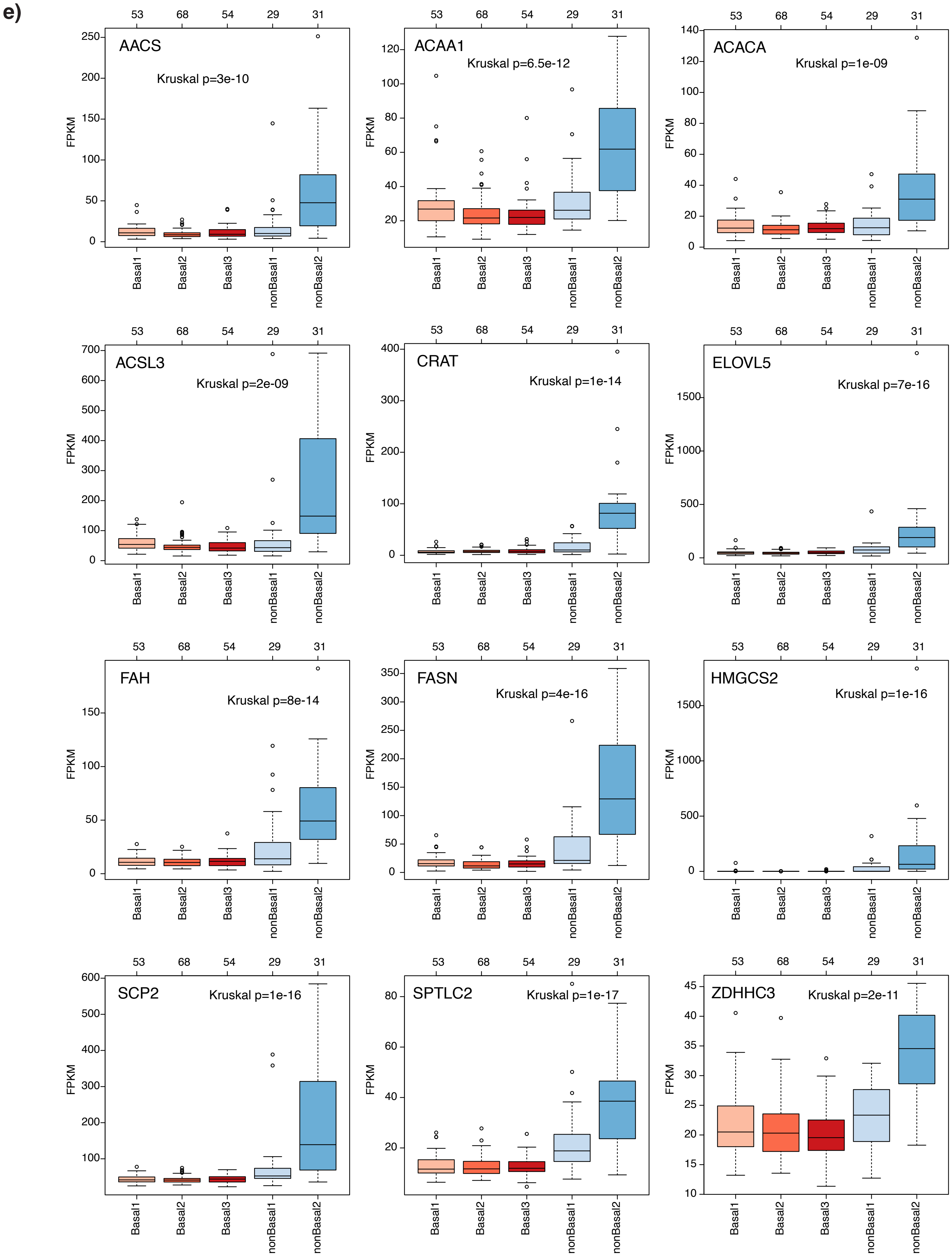

Supplementary Figure S6

f)

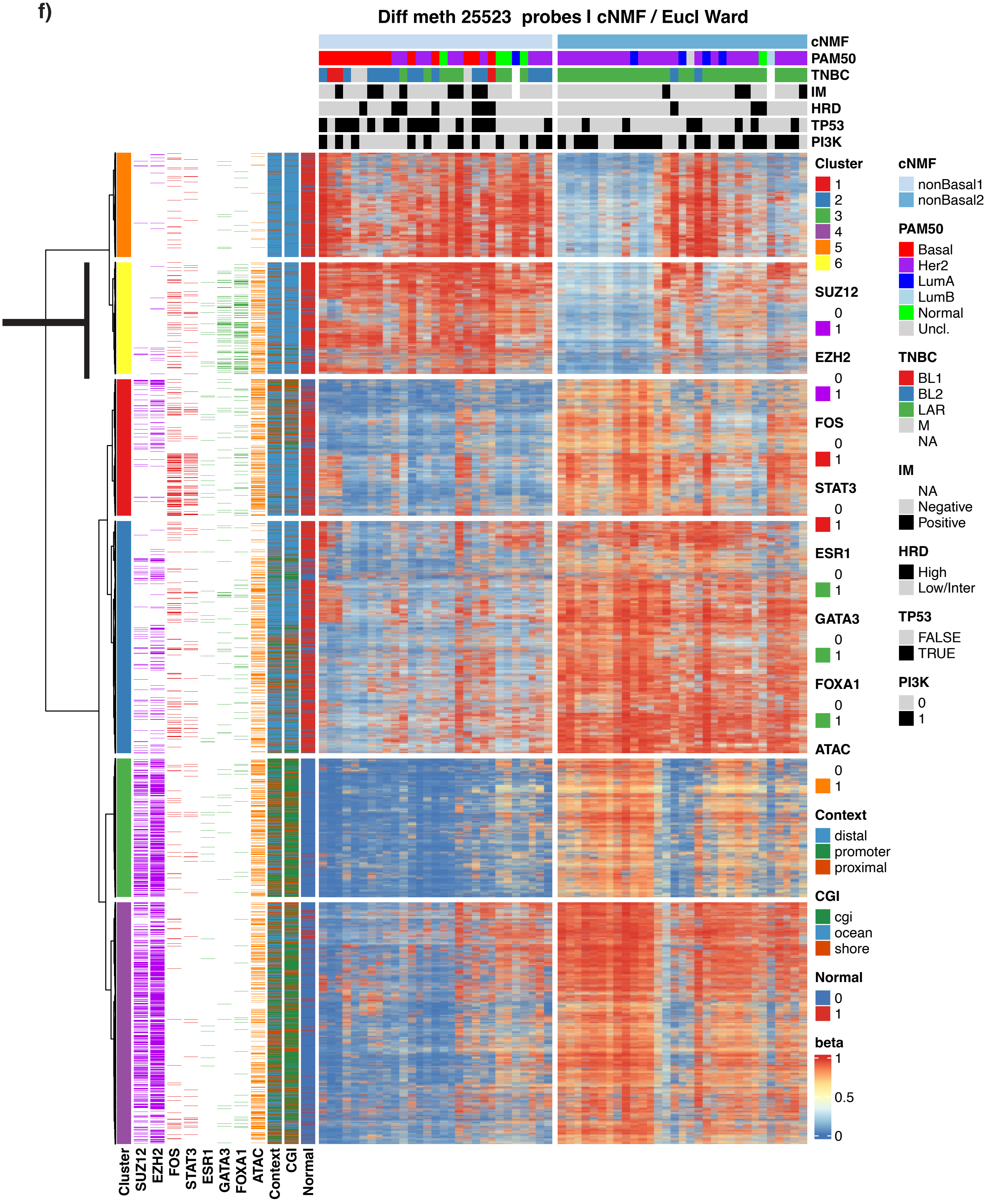

Supplementary Figure S6

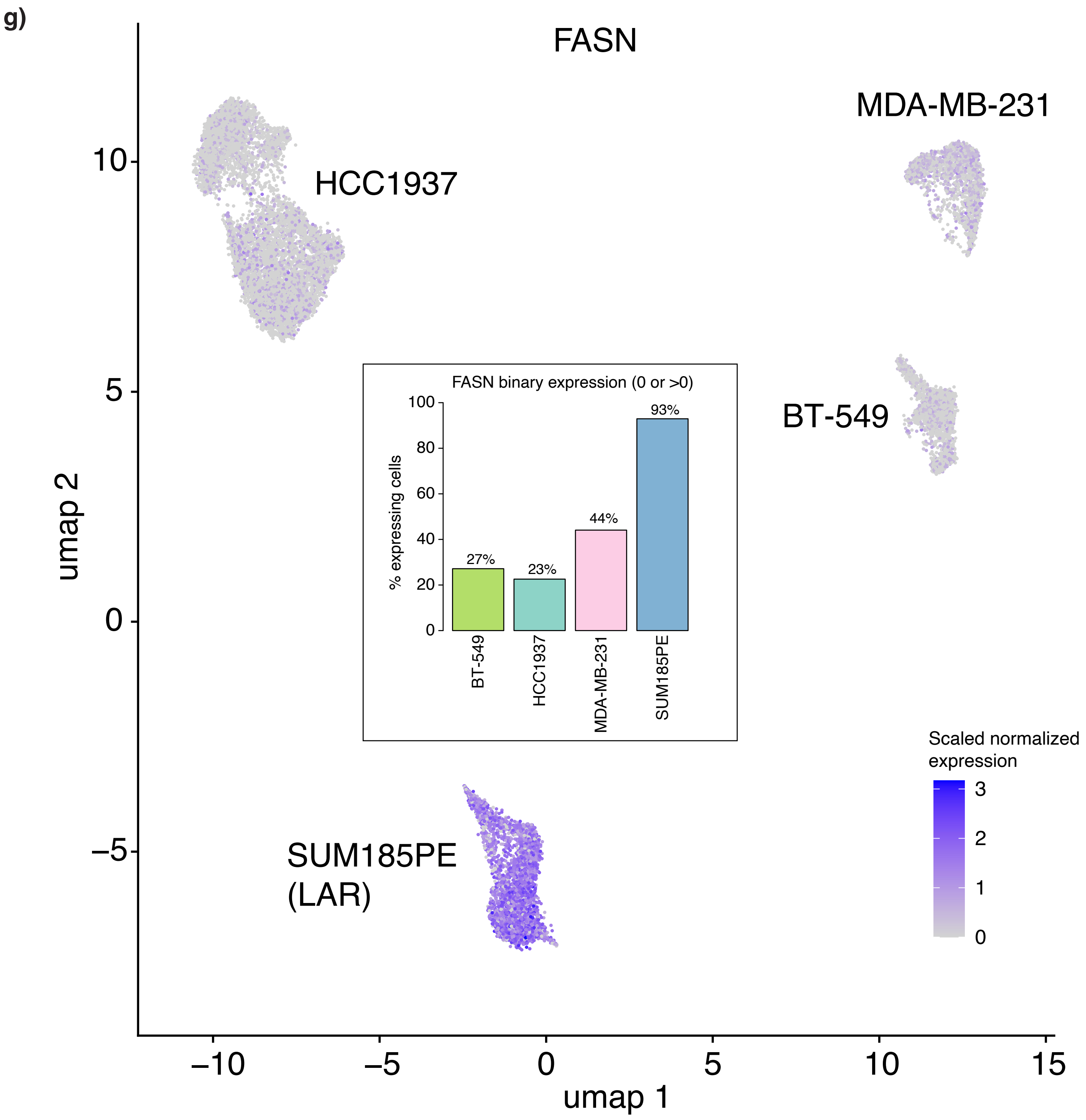

Supplementary Figure S6

h)

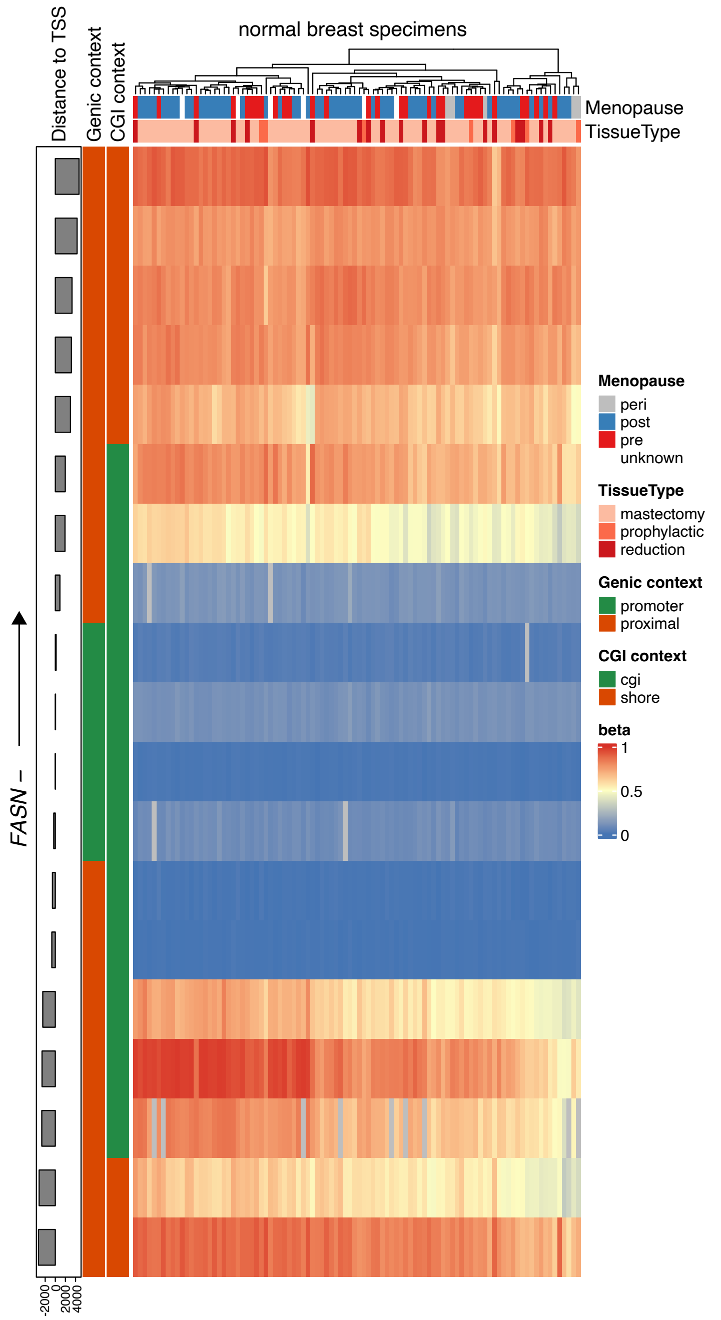

i)

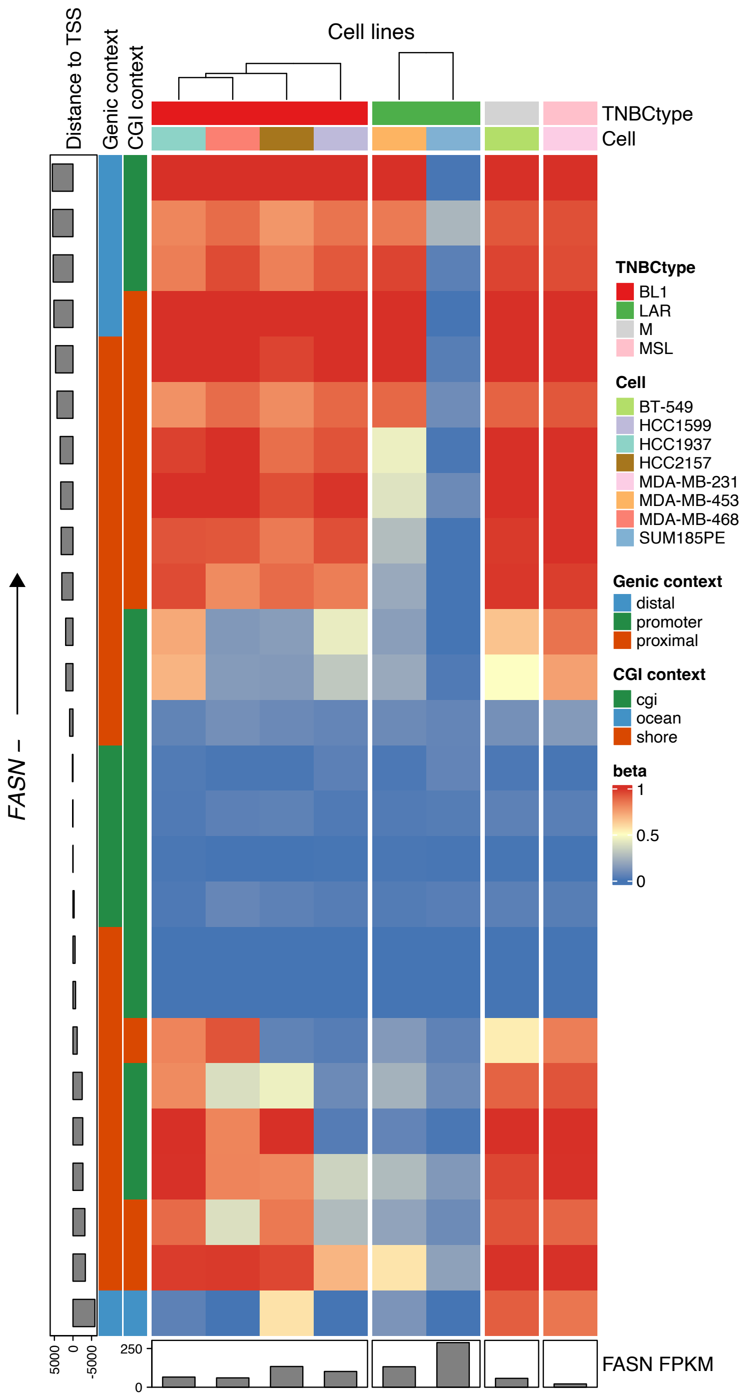

Supplementary Figure S6

j)

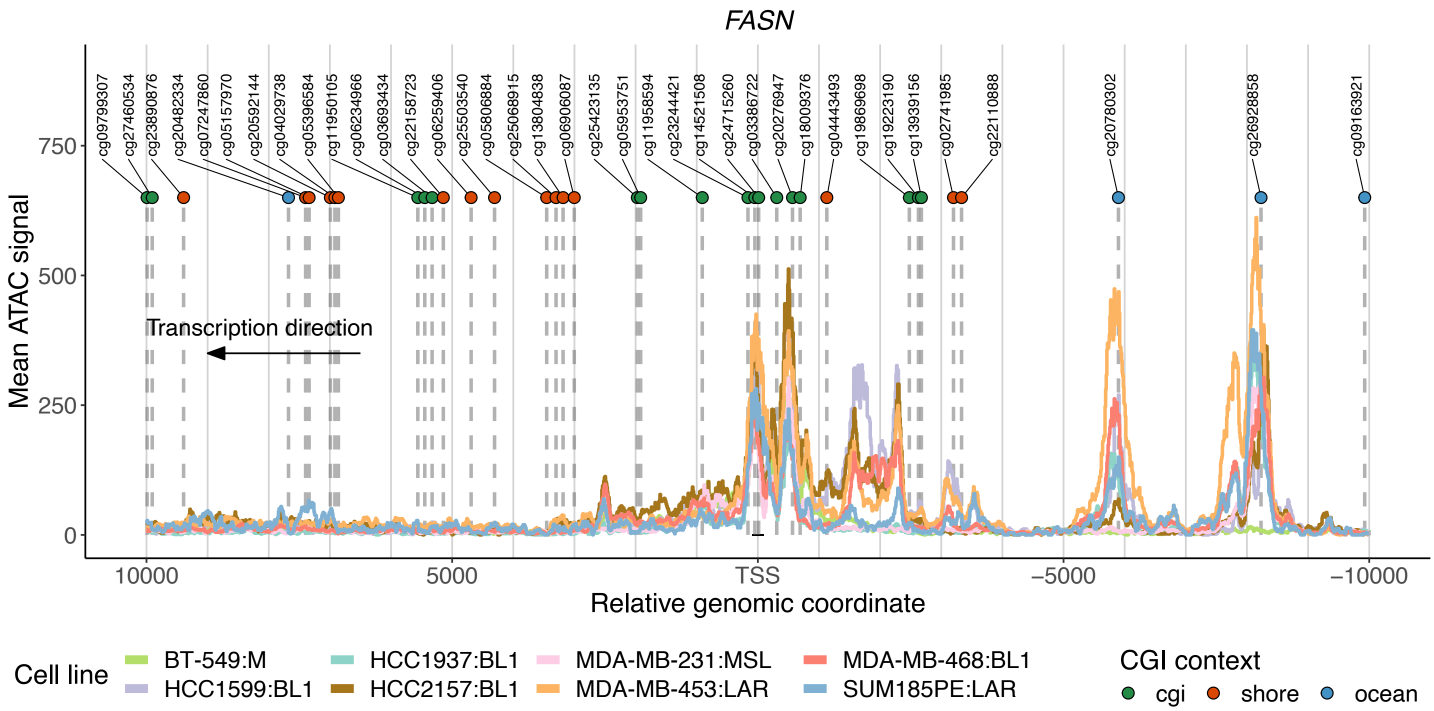

Supplementary Figure S6

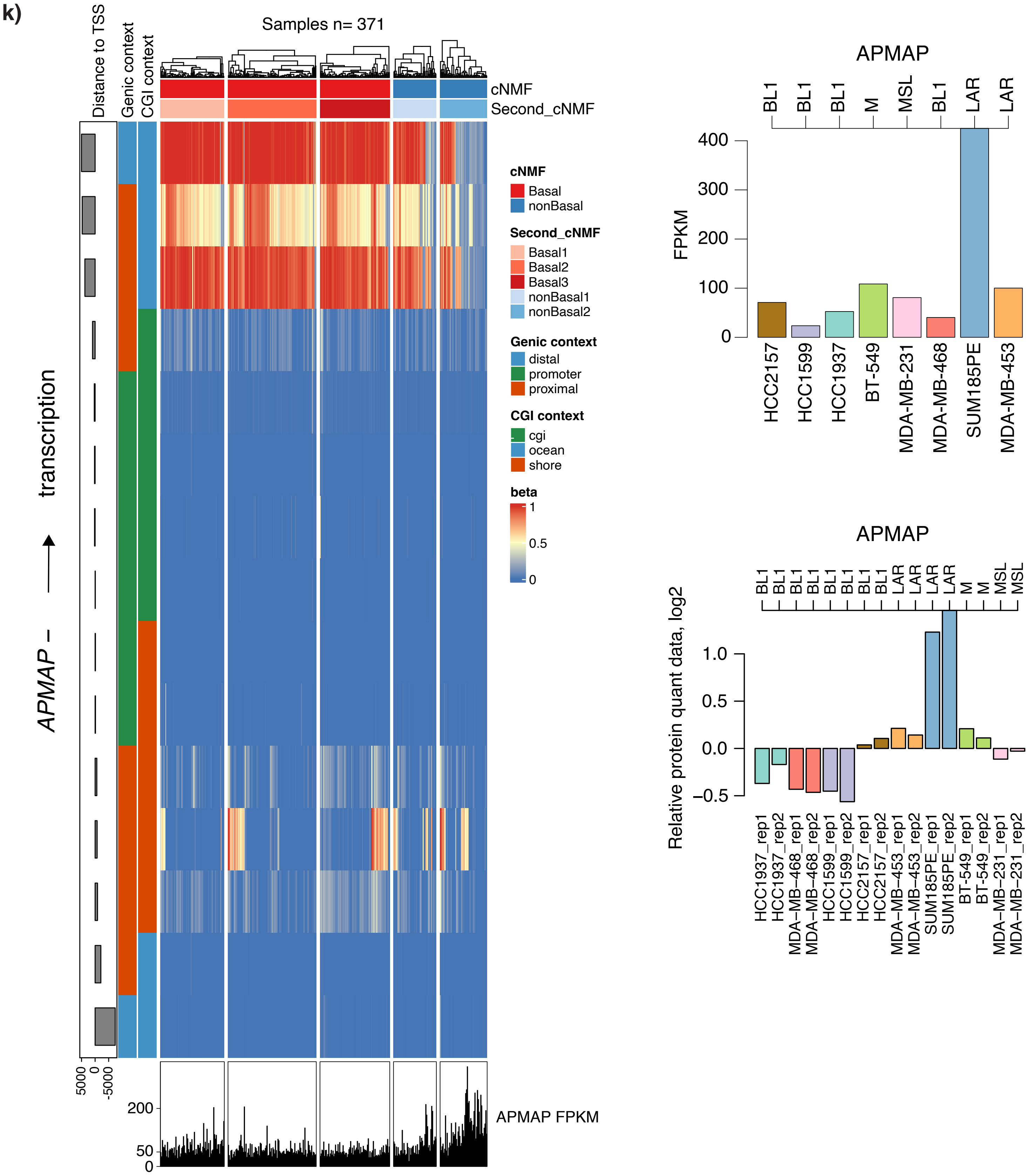

Supplementary Figure S6

I)

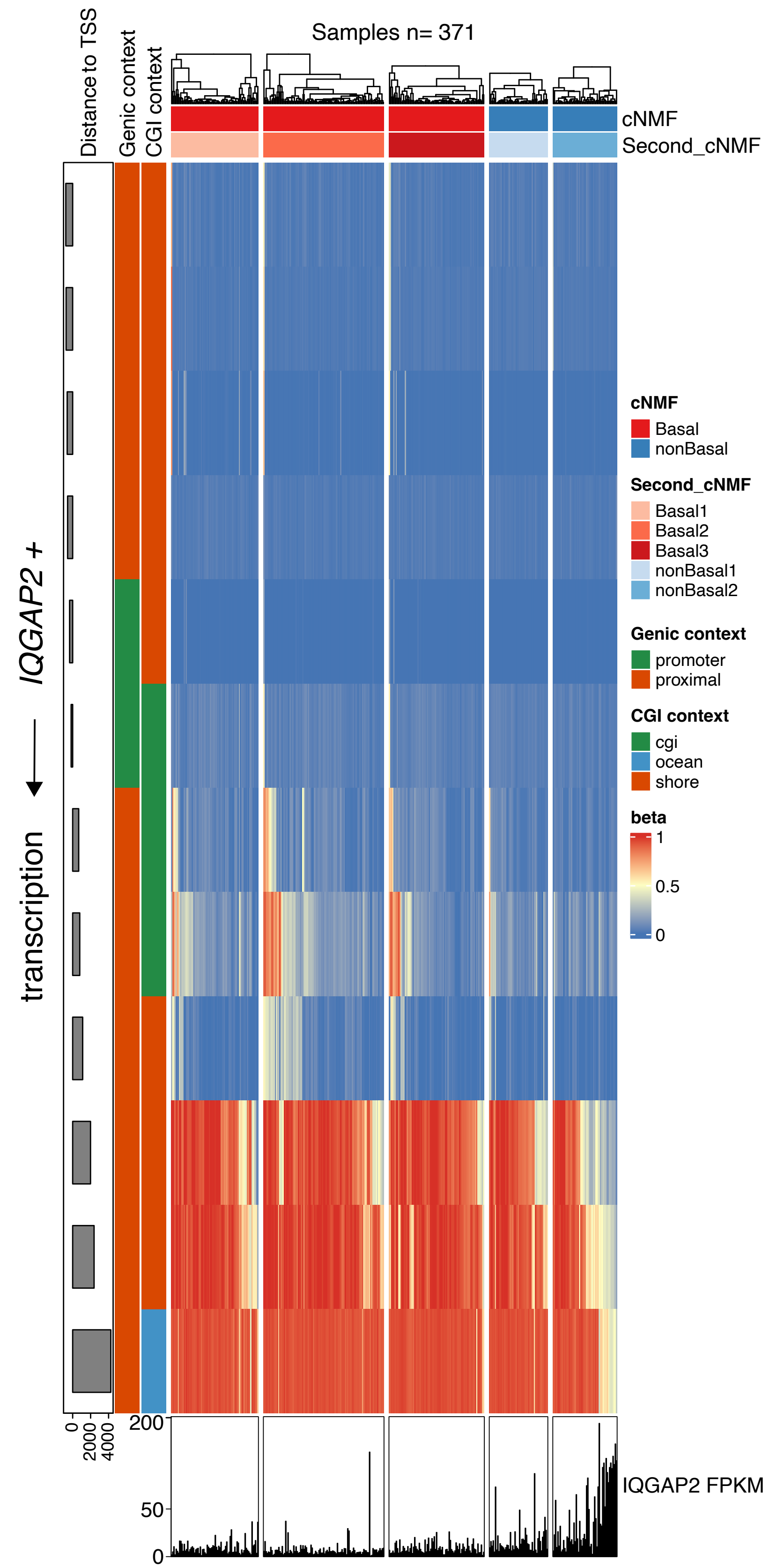

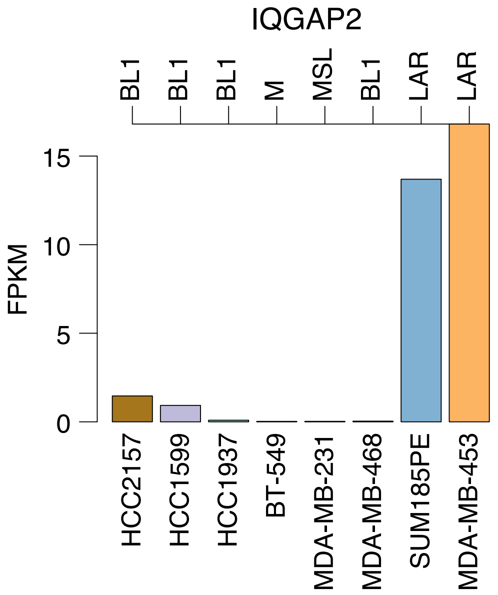

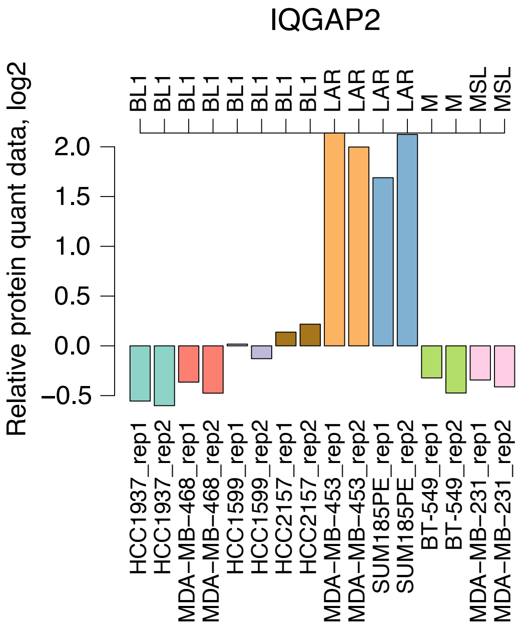

Supplementary Figure S6. Characteristics of the nonBasal1 and nonBasal2 subgroups. (a) ASCAT purity estimates for the nonBasal subgroups. **(b)** Homologous recombination deficiency (HRD) proportions for the nonBasal subgroups. **(c)** Scores of 8 metagenes for the nonBasal subgroups and androgen receptor (*AR*) mRNA expression. P-values computed using Wilcoxon's test. **(d)** Heatmap of scaled expression for 137 network3 genes with high FPKM expression in SCAN-B tumors in single cell data from the TNBC study by Wu et al. (PMID:32790115) using the Broad Single Cell Portal (PMID:37502904). Dot size corresponds to % expressing cells for a cell type. Analyses were performed using default web portal parameters and settings. **(e)** FPKM expression plots of genes included in the metabolism GAM plot in Figure 7f, stratified by Basal and nonBasal subgroups in the 235 SCAN-B tumors. P-value calculated using Kruskal-Wallis test. **(f)** Extended differential methylation analysis between nonBasal1 and nonBasal2 SCAN-B tumors. 25523 CpGs selected based on an absolute median beta value difference between groups >0.5 without a p-value requirement. CpG row cluster 6 (yellow, highlighted) is strongly enriched for FOXA1/GATA3 binding site overlaps. Clustering was performed using Euclidian distance and Ward.D linkage. **(g)** UMAP plot of single cell RNAseq (scRNAseq) data for four TNBC cell lines showing normalized and scaled expression of *FASN*. Inserted bar plot shows the proportion of cells with a binary expression of the gene (0 expression or >0) per cell line. **(h)** Promoter methylation for *FASN* in 96 normal breast specimens. CpGs ordered according to increasing genomic coordinate. *FASN* strand direction is minus (-). **(i)** Promoter methylation for *FASN* in 8 TNBC cell lines ordered by TNBCtype. CpGs ordered according to increasing genomic coordinate. *FASN* strand direction is minus (-). **(j)** Mean ATAC-seq signal along the *FASN* promoter region for the 8 TNBC cell lines. CpG positions and their CGI context are superimposed. TSS = transcription start site. **(k)** Promoter methylation for *APMAP*, included in the nonBasal2 metabolism gene network. Right bar plots show mRNA (FPKM) and relative protein expression (2 replicates per cell line showed) in the same TNBC cell lines. **(l)** Promoter methylation for *IQGAP2*, included in the nonBasal2 metabolism gene network. Right bar plots show mRNA (FPKM) and relative protein expression (2 replicates per cell line showed) in the TNBC cell lines.

All reported p-values from statistical tests are two-sided if not otherwise specified. Boxplot elements correspond to: (i) center line = median, (ii) box limits = upper and lower quartiles, (iii) whiskers = 1.5x interquartile range. Top-axis in boxplots reports group sizes.

Supplementary Figure S7

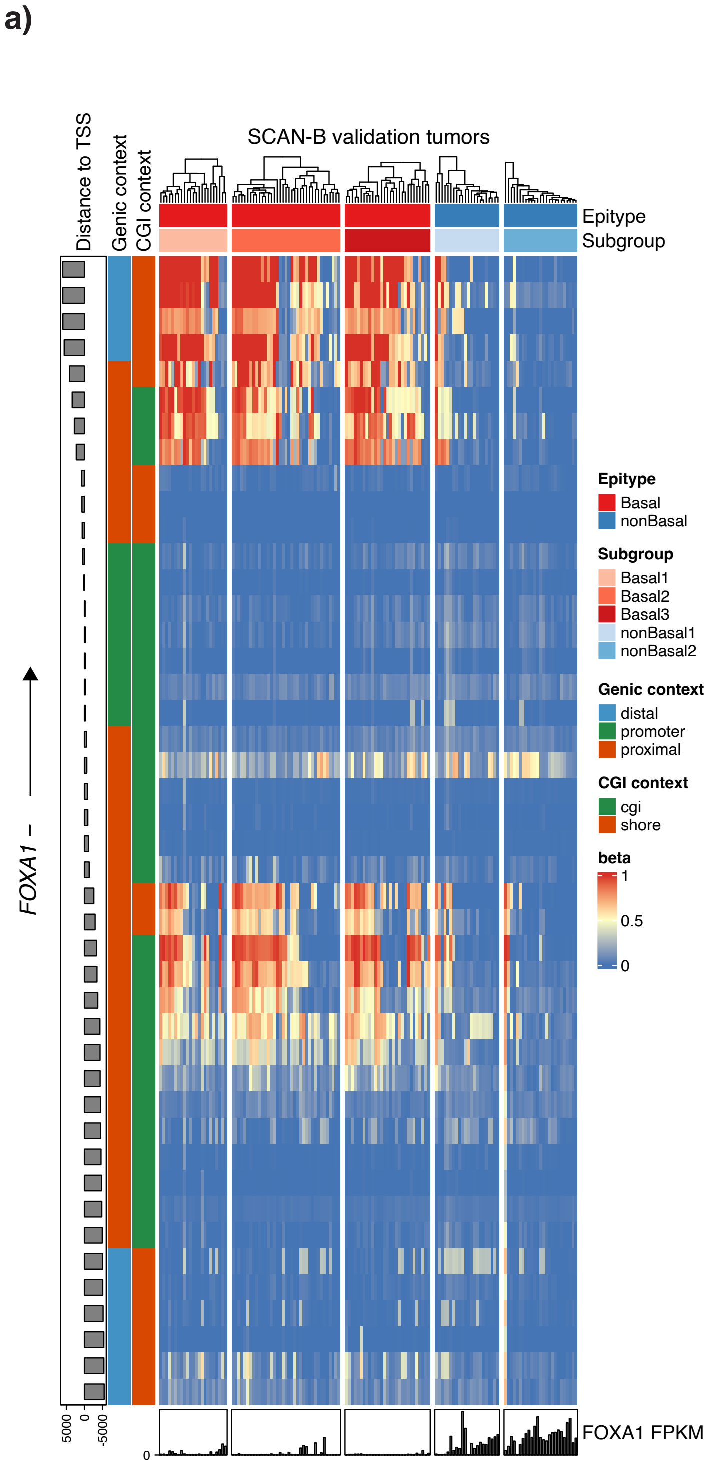

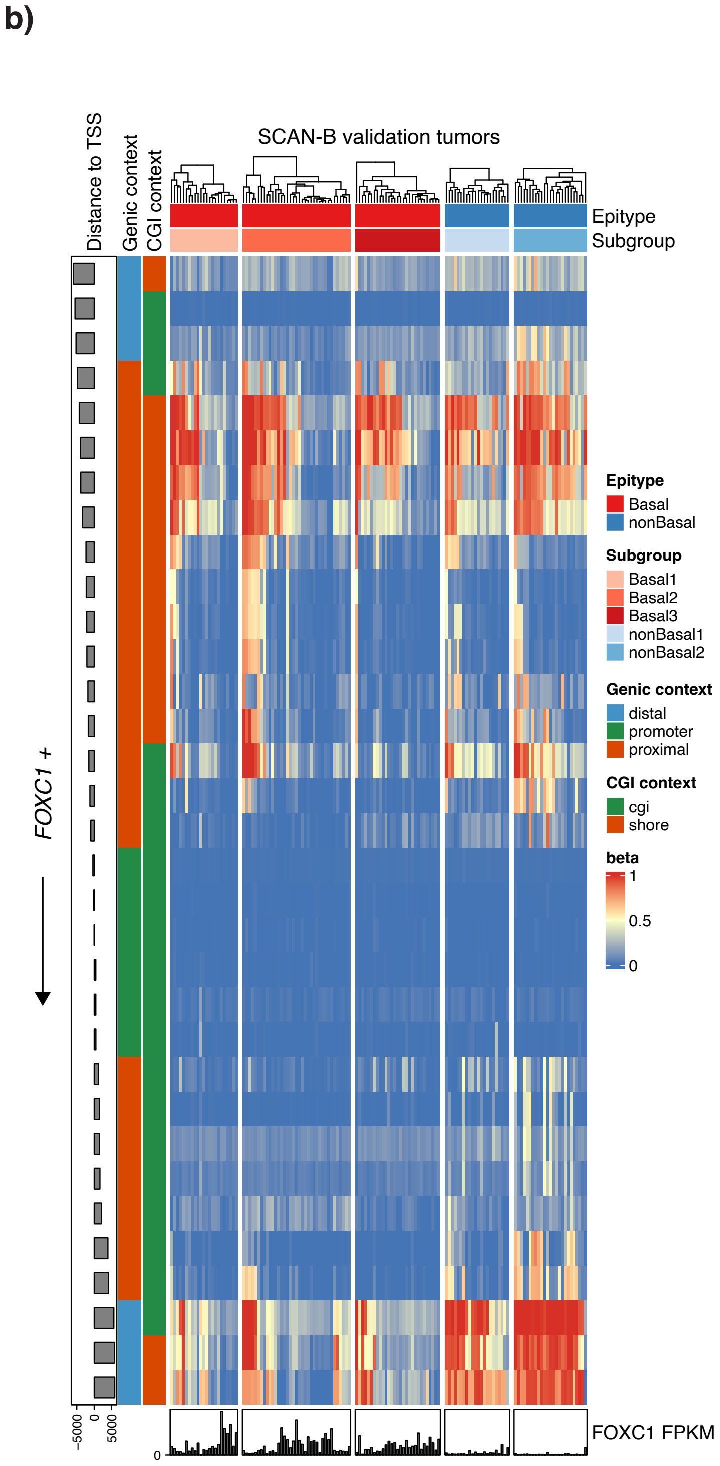

Supplementary Figure S7

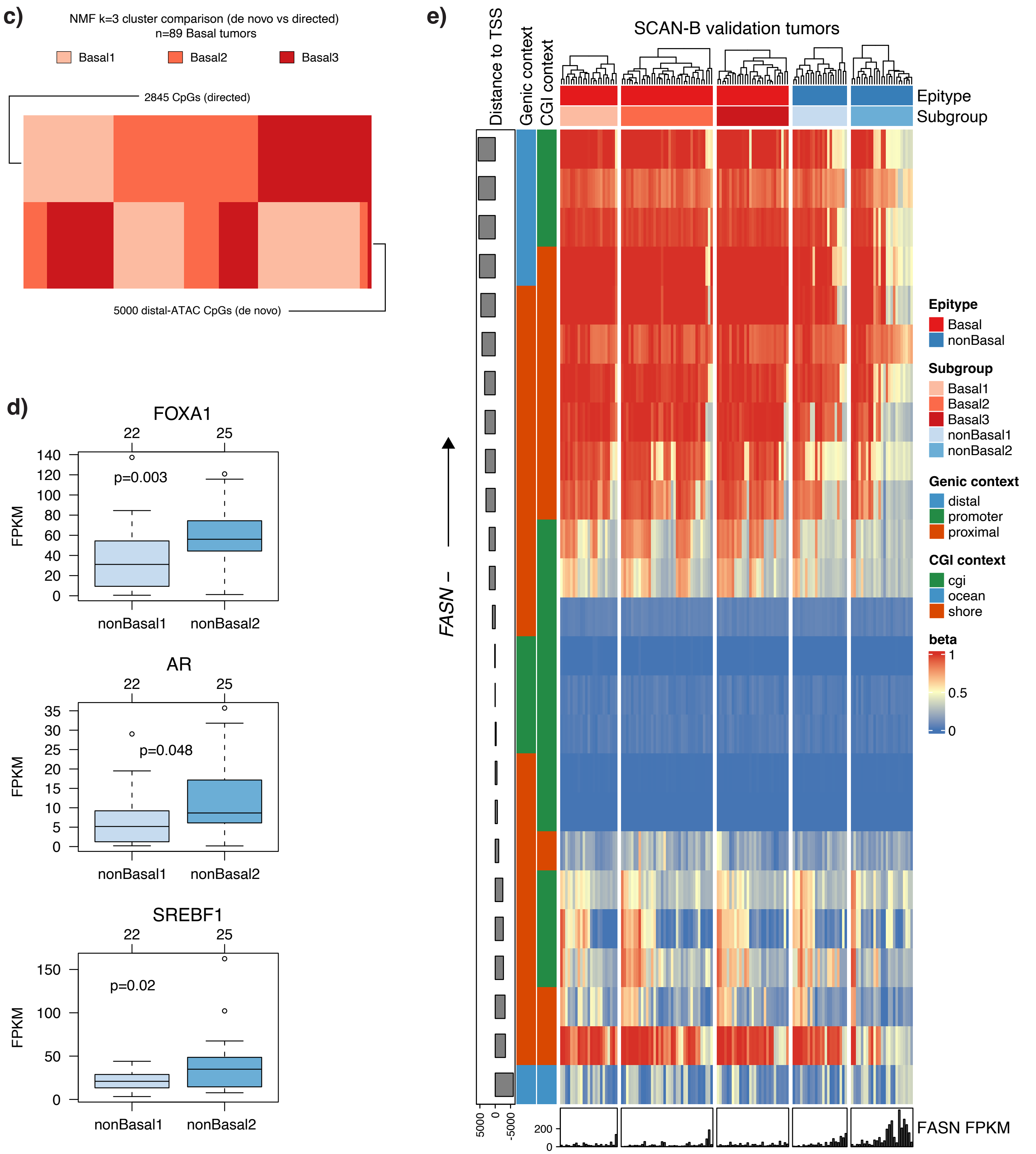

Supplementary Figure S7. Analyses in the SCAN-B TNBC 136-sample validation cohort. (a) *FOXA1* promoter methylation plot for the SCAN-B validation cohort. CpGs are ordered in increasing base pair position. *FOXA1* strand direction is minus (-). Bottom panel shows *FOXA1* FPKM levels. **(b)** *FOXC1* promoter methylation plot for the SCAN-B validation cohort. CpGs are ordered in increasing base pair position. *FOXC1* strand direction is minus (-). Bottom panel shows *FOXC1* FPKM levels. **(c)** Agreement between three-group (k=3) NMF cluster solutions in 89 Basal tumors, based on either a de novo analysis using the 5000 most variant distal-ATAC CpGs in Basal tumors or based on the 2845 CpGs differentially methylated between Basal1-3 tumors in the SCAN-B discovery cohort (directed CpG set). **(d)** mRNA expression (FPKM) of 3 genes for nonBasal subgroups. Two-sided p-values are calculated using Wilcoxon's test. **(e)** *FASN* promoter methylation plot for the SCAN-B validation cohort. CpGs are ordered in increasing base pair position. *FASN* strand direction is minus (-). Bottom panel shows *FOXA1* FPKM levels.
All reported p-values from statistical tests are two-sided if not otherwise specified. Boxplot elements correspond to: (i) center line = median, (ii) box limits = upper and lower quartiles, (iii) whiskers = 1.5x interquartile range. Top-axis in boxplots reports group sizes.

Supplementary Figure S8

a)

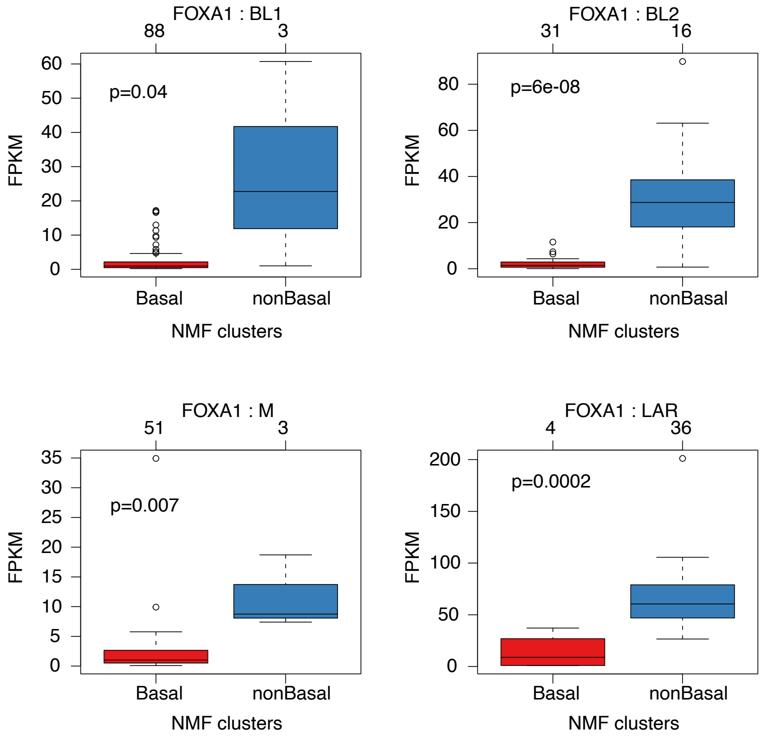

b)

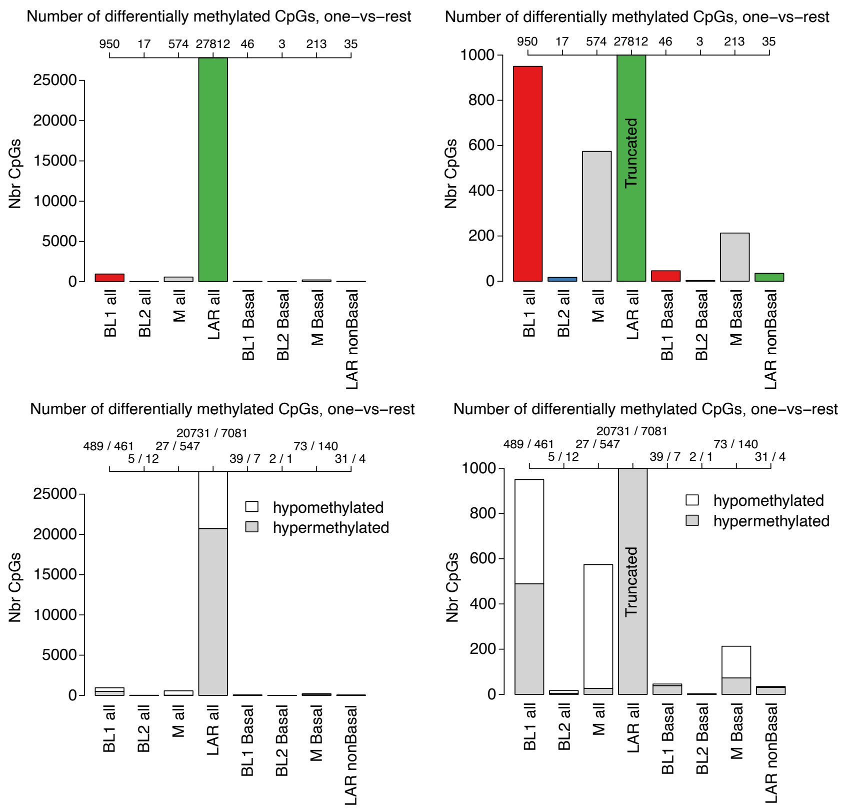

Supplementary Figure S8. mRNA and epigenetic patterns for TNBCtype subtypes. (**a**) Boxplots of *FOXA1* FPKM expression for TNBCtype subtypes divided by Basal and nonBasal epitypes. P-values computed using Wilcoxon's test. (**b**) Number of differentially methylated CpGs for TNBCtype mRNA subtypes identified using a "one versus rest" analysis in all SCAN-B discovery cohort tumors (all), or Basal tumors only (Basal), or nonBasal tumors only (nonBasal). Only groups with >1 CpG identified are shown. Significant CpGs identified using Bonferroni-adjusted Wilcoxon's test, p<0.01, absolute beta delta >0.25. Top plots show total number of differentially methylated CpGs, bottom plots show number of significant CpGs stratified by whether they are hypomethylated or hypermethylated in the tested group. Plots on the right are zoomed in versions of the plots on the left.
All reported p-values from statistical tests are two-sided if not otherwise specified. Boxplot elements correspond to: (i) center line = median, (ii) box limits = upper and lower quartiles, (iii) whiskers = 1.5x interquartile range. Top-axis in boxplots reports group sizes.
