# Supplementary material for: The DNA methylation landscape of primary triple-negative breast cancer
Source: Nat Commun. 2025 Mar 28;16:3041. doi: 10.1038/s41467-025-58158-x (PMC11953470; doi:10.1038/s41467-025-58158-x)
Supplement: Supplementary file 2 — Description of Additional Supplementary Information [file 41467_2025_58158_MOESM2_ESM.pdf]

Description of Additional Supplementary Files

Supplementary Data 1

Description: Clinicopathological characteristics of the SCAN-B discovery and validation cohorts. Two main tables are included: (1a) containing information on the SCAN-B discovery cohort (column header legends are in a different table); (1b) containing information on the SCAN-B validation cohort (column header legends are in a different table).

Supplementary Data 2

Description: CpG enrichments, differentially methylated CpGs, significantly correlated CpG-gene pairs, and candidate epigenetically regulated genes for Basal vs nonBasal tumors in the SCAN-B discovery cohort. Four tables are included: (2a) transcription factor enrichment for CpG row clusters; (2b) differentially methylated CpGs between Basal and nonBasal NMF clusters; (2c) significant CpG-gene correlations related to differentially methylated CpGs; (2d) candidate genes for epigenetic regulation Basal/nonBasal.

Supplementary Data 3

Description: Pathway analysis of specific differentially expressed genes for the Basal1-3 groups. Three main tables are included: (3a) specific differentially expressed genes for Basal1, Basal2, and Basal3 NMF clusters (a different table for each cluster); (3b) pathway enrichment results for differentially expressed genes in 3a (different tables for KEGG, GO MF, Hallmark, and Reactome pathways for Basal1 cluster, then two different tables for Hallmark pathways in Basal2 and Basal3); (3c) significantly correlated CpG-gene pairs in tumors and TNBC cell lines.

Supplementary Data 4

Description: Differential methylation and mRNA expression for the nonBasal subdivision in SCAN-B tumors. Six main tables are included: (4a) differentially methylated CpGs between nonBasal 1 and nonBasal2 NMF clusters; (4b) differentially expressed genes between nonBasal1 and nonBasal2 NMF clusters; (4c) pathway enrichment results for differentially expressed genes in 4b (different tables for KEGG, GO MF, Hallmark, and Reactome pathways); (4d) Cytoscape networks 1-3; (4e) pathway enrichment results for Cytoscape networks; (4f) significant CpG-gene pairs with negative correlation and close to a TSS for nonBasal tumors.

Supplementary Data 5

Description: Raw and normalized proteogenomic data for 8 TNBC cell lines. Four tables are included: Legends, containing explanations for columns found in the other tables; Sample Info, containing information on each cell line replicate so sample columns can be properly identified; All Data, containing protein information and quantification in each sample; Quant. Data, containing the final normalized values per sample per protein.
