# Supplementary material for: The DNA methylation landscape of primary triple-negative breast cancer
Source: Nat Commun. 2025 Mar 28;16:3041. doi: 10.1038/s41467-025-58158-x (PMC11953470; doi:10.1038/s41467-025-58158-x)
Supplement: Supplementary file 8 — Reporting Summary [file 41467_2025_58158_MOESM8_ESM.pdf]

nature portfolio

Corresponding author(s): Johan Staaf

Last updated by author(s): Mar 3, 2025

Reporting Summary

Nature Portfolio wishes to improve the reproducibility of the work that we publish. This form provides structure for consistency and transparency in reporting. For further information on Nature Portfolio policies, see our Editorial Policies and the Editorial Policy Checklist.

Statistics

For all statistical analyses, confirm that the following items are present in the figure legend, table legend, main text, or Methods section.

n/a	Confirmed	
☐	☒	The exact sample size (*n*) for each experimental group/condition, given as a discrete number and unit of measurement
☐	☒	A statement on whether measurements were taken from distinct samples or whether the same sample was measured repeatedly
☐	☒	The statistical test(s) used AND whether they are one- or two-sided<br>*Only common tests should be described solely by name; describe more complex techniques in the Methods section.*
☐	☒	A description of all covariates tested
☐	☒	A description of any assumptions or corrections, such as tests of normality and adjustment for multiple comparisons
☐	☒	A full description of the statistical parameters including central tendency (e.g. means) or other basic estimates (e.g. regression coefficient) AND variation (e.g. standard deviation) or associated estimates of uncertainty (e.g. confidence intervals)
☐	☒	For null hypothesis testing, the test statistic (e.g. *F*, *t*, *r*) with confidence intervals, effect sizes, degrees of freedom and *P* value noted<br>*Give P values as exact values whenever suitable.*
☒	☐	For Bayesian analysis, information on the choice of priors and Markov chain Monte Carlo settings
☒	☐	For hierarchical and complex designs, identification of the appropriate level for tests and full reporting of outcomes
☐	☒	Estimates of effect sizes (e.g. Cohen's *d*, Pearson's *r*), indicating how they were calculated

Our web collection on statistics for biologists contains articles on many of the points above.

Software and code

Policy information about availability of computer code

Data collection	Data collection sources are outlined in the Data Availability Statement<br><br>For SCAN-B patients:<br>RNA-sequencing for included patients was obtained from online repository associated with Staaf et al. NPJ Breast Cancer 2022.<br><br>Processed somatic WGS data was collected from an online repository associated with the Study by Staaf et al. Nat Med 2019.<br><br>DNA methylation analysis of SCAN-B TNBC discovery cohort<br>DNA methylation analysis was performed on the same DNA used for WGS using Illumina EPIC v1 beadchips according to manufacturer's instructions for 235 tumors. Beta values, representing the level of methylation, were computed using the minfi R package v1.44 function preprocessFunnorm() and Infinium probe normalized using the approach described by Holm et al. BCR 2016.<br><br>DNA methylation of the SCAN-B TNBC validation cohort<br>To validate DNA methylation patterns and subgroups derived from the discovery SCAN-B cohort, a set of 136 non-overlapping SCAN-B TNBC tumors part of the cohort reported by Staaf et al. NPJ Breast cancer 2022 was analyzed using MethylationEPIC v1.0 or v2.0 BeadChip according to manufacturer's instructions by the SNP&SEQ Technology Platform in Uppsala, Sweden (www.genotyping.se).<br><br>The Cancer Genome Atlas (TCGA)<br>Processed and compiled DNA methylation data (Illumina 450K beadchips) and RNA-sequencing FPKM estimates for 645 breast cancers with matched data from the TCGA cohort was obtained from the study by Staaf and Aine Plos One 2022. DNA methylation data (beta-values) were

adjusted for tumor cell content as described in Staaf and Aine Plos One 2022 for each tumor. TCGA tumors were PAM50 subtyped using the NCN approach described by Staaf et al. NPJ Breast Cancer 2022 , using the same reference sets and code.

Data analysis

Additional detailed data is available in the supplementary methods document associated with the submission.
All analyses and associated statistical tests were performed using the R programming language.

RNA-sequencing SCAN-B
RNA-sequencing was performed by Illumina stranded TruSeq mRNA protocol, either implemented on KingFisher or on the Illumina NeoPrep system. Expression data (Fragments Per Kilobase per Million reads, FPKM) from stringtie was derived from RNA-sequencing data using an analysis pipeline to align and estimate gene expression values for sequenced samples. The RNA-sequencing analysis pipeline is based on a collection of open source software tools; picard tools, trimmomatic, bowtie2, hisat2, stringtie with the GRCh38 human genome primary assembly, dbSNP, and GENCODE transcriptome model as detailed in Supplemental methods in the publication by Staaf et al. NPJ Breast Cancer 2022. Entrez ID from the Gencode27 metadata was used as gene identifiers.

DNA methylation annotation, clustering, and differential methylation analysis
Processed CpG beta values were corrected for tumor purity using the approach described by Staaf and Aine Plos One 2022 using tumor cell content estimations from WGS with the ASCAT software obtained from the repository associated with Staaf et al. NatMed 2019. After basic data processing and CpG beta correction, 760405 CpGs remained for analysis, referred to as purity-adjusted beta values. These were further filtered to exclude chrX/Y localization and non-CpG probes, leaving 741145 CpGs for analyses. Clustering of DNA methylation data was performed using Non-negative Matrix Factorization (NMF) through the R package NMF v0.28 27. Heatmaps were produced using the pheatmap v1.0.12 or the ComplexHeatmap v2.20.0 R packages. Details of the beta value correction, NMF clustering, differential beta value methylation analysis, and correlation of CpG beta values to gene expression data are provided in the Supplementary Methods. We compiled a custom feature annotation set for each probe on the Illumina EPIC methylation platform using the same methodology described for the Illumina HumanMethylation450K array in Staaf and Aine Plos One 2022. This included assigning CpGs to a gene-centric context defined as “promoter” (+/- 500 bp centered on gene transcription start site, TSS), “proximal” (+/- 5 kbp centered on but excluding and excluding the promoter window), or “distal” (>5 kbp from TSS) based on their genomic coordinates (referred to as “genic context”). For the gene-centric annotations, a consensus transcript model based on GENCODE v27 protein coding genes matching SCAN-B RNA-seq data was built for each gene by collapsing of exons. The 5’ most base was assigned as the consensus TSS and the 3’ most base as the consensus transcription termination site. For the TCGA cohort profiled on Illumina 450K, gene annotations were derived using the same method except the transcript models were based on the GENCODE 22 TCGA reference file “gencode.gene.info.v22.tsv” matching the TCGA RNA-seq data (https://gdc.cancer.gov/about-data/gdc-data-processing/gdc-reference-files). For global analyses, probes were assigned a consensus category based on the hierarchy: i) localization in a gene promoter, ii) localization in a proximal region, iii) distal was defined as neither i) or ii). Probes were also assigned to a CpG-centric context defined as “CpG island” (CGI), “shore”, or “ocean” (referred to as “CGI context”) 22. Local CpG density metrics (e.g., O/E) and contextual classifications for each probe were obtained using the methods of Saxonov et al. for high (HCG) and low (LCG) CpG content and of Weber et al. for HCP, ICP, and LCP. EPIC probe overlaps with ATAC-seq peak data generated on 74 TCGA breast cancer samples by Corces et al. were calculated and used as a proxy for differentially open chromatin in breast cancer. Additionally, ENCODE candidate cis-regulatory elements and ENCODE ChIP-seq peak overlaps for 340 transcription factors in 130 cell lines were used to assess the regulatory potential of EPIC CpGs.

Gene expression analyses
Gene expression profiling was performed using RNA-sequencing (RNAseq) and has been reported previously as fragments per kilobase per million reads (FPKM) values (see Staaf et al. NPJ Breast Cancer). PAM50 classification was obtained from Staaf et al. NPJ Breast Cancer. Other transcriptional classifications, including the TNBCtype-4 subtypes 6, were obtained as described (Staaf et al. Nat Med, Glodzik et al. Nat Com), and a TNBCtype IM class as detailed in the Supplementary Methods. Cell type deconvolution results were obtained from Glodzik et al. for CIBERSORTx. Based on FPKM data, gene expression-based rank scores for eight biological metagenes in breast cancer originally defined by Fredlund et al. (BCR 2012) were calculated as described in Nacer et al. (Genome Med 2023). Rank scores were computed individually for each tumor from FPKM data without any further normalization or data centering. Differential gene expression analysis and pathway analysis were performed as described in the Supplementary Methods.

Whole genome sequencing analyses
WGS data, including mutational calls, mutational and rearrangement signatures, copy number profiles, and HRD classification by HRDetect 13 were obtained from Staaf et al. Nat Med 2019.

PD-L1 immunohistochemistry and tumor infiltrating lymphocytes (TILs)
PD-L1 was assessed immunohistochemically using the 22C3 clone on a Dako Autostainer Link 48 platform (Agilent, Inc., CA, U.S.) in formalin-fixed, paraffin-embedded tumor samples in a tissue microarray (TMA) where each sample was represented by two TMA cores with a diameter of 1 mm (TMA construction outlined in Aine et al. BCR 2021. Preparations and stainings were done according to the manufacturer’s instructions. PD-L1 expression was evaluated as tumor positive score (TPS) in percentage as described in the manual for the PD-L1 IHC 22C3 pharmDx antibody (Agilent). The TMA core with the highest score was set as the score for the respective tumor. Of all 235 tumors, 207 could be evaluated. Additionally, TIL estimations (% TILs) were obtained from hematoxylin and eosin-stained whole slides from Aine et al. BCR 2021 for 211 cases.

TNBC cell lines and genomic analyses
Eight TNBC cell lines proposed to represent different TNBCtype mRNA subtypes (HCC2157:BL1, HCC1599:BL1, HCC1937:BL1, BT-549:M, MDA-MB-231:MSL, MDA-MB-468:BL1, SUM185PE:LAR, and MDA-MB-453:LAR (Lehmann et al. JCI 2011) were obtained from commercial or in-house biobanks and used for ATAC-sequencing (ATAC-seq), RNAseq, DNA methylation, and proteomic analysis. Cell lines were grown as detailed in the Supplementary Methods and DNA, RNA, and a protein flow-through fraction were extracted using the Qiagen Allprep DNA/RNA mini kit (cat no 80204) following manufacturer’s instructions. ATAC-seq was performed and analyzed on each cell line as outlined by Arbajian et al.. RNAseq libraries were prepared using the Illumina TruSeq stranded mRNA protocol and sequenced on the NovaSeq 6000 system at the Center for Translational Genomics (www.ctg.lu.se) in Lund, Sweden. Demultiplexing was performed using the bcl2fastq2 software (Illumina) with default settings and the quality was checked with FastQC. Reads were mapped to the GRCh38 reference genome using the HISAT2 software and annotation files from release 103. Finally, expression data in FPKM were calculated with StringTie. DNA methylation data were generated for each cell line using the Illumina Infinium MethylationEPIC v1.0 BeadChip (interrogating ~800,000 CpGs) according to manufacturer’s instructions by the SNP&SEQ Technology Platform in Uppsala, Sweden (www.genotyping.se). Basic processing of beadchip

data was performed as described (Staaf and Aine, Plos One 2022). Global proteomics was performed using mass spectrometry for all eight cell lines as detailed in the Supplementary Methods by the Clinical Proteomics Mass Spectrometry facility, Karolinska Institutet/Karolinska University Hospital/Science for Life Laboratory, Solna, Sweden.

SCAN-B TNBC validation cohort
To validate DNA methylation patterns and subgroups derived from the discovery SCAN-B cohort, a set of 136 non-overlapping SCAN-B TNBC tumors part of the cohort reported by Staaf et al. NPJ Breast Cancer 2022 was analyzed using MethylationEPIC v1.0 or v2.0 BeadChip according to manufacturer's instructions by the SNP&SEQ Technology Platform in Uppsala, Sweden (www.genotyping.se). This patient set is hereon referred to as the SCAN-B validation cohort. Matched clinicopathological and RNAseq FPKM data, including PAM50 subtypes, were obtained from Staaf et al. NPJ Breast Cancer 2022. TNBCtype classification was performed as described in the Supplementary Methods. Basic DNA methylation processing was performed as described in the Supplementary Methods. In the absence of tumor purity estimates from WGS, we used the PureBeta pipeline to estimate tumor purity directly from DNA methylation data. Next, estimated tumor purities were combined with CpG models derived in the SCAN-B discovery cohort by the Staaf and Aine method to adjust beta values through the PureBeta pipeline (see Supplementary Methods for details). After all processing steps, 701304 CpGs remained for analysis. Patient specific data for the validation cohort is available in Supplementary Table S1b.

Multi-omics TCGA general breast cancer validation cohort
Matched DNA methylation data from Illumina 450K bead arrays, RNAseq FPKM data and whole exome sequencing (WES) somatic mutation data for 645 breast cancers of all clinical subgroups from the TCGA consortium were obtained and processed as described in Staaf and Aine Plos One 2022. For DNA methylation data, 381355 CpGs remained after annotation mapping, filtering and EPIC platform matching. TCGA tumors were PAM50 subtyped using the nearest centroid classification approach described by Staaf et al. NPJ Breast Cancer 2022, using the same reference sets and code to ensure single sample classification.

Non-overlapping general breast cancer SCAN-B RNAseq cohort
RNAseq FPKM data from 6233 primary tumors were collected as described by Veerla et al. 39 from data deposited by Staaf et al. NPJ Breast Cancer 2022. Of the 6233 tumors, 6009 did not overlap with the 235-sample SCAN-B TNBC discovery cohort and were used to contrast gene networks in a general breast cancer population. All patient and tumor annotations were taken from Staaf et al. NPJ Breast Cancer 2022. RNAseq processing is identical for the 6009 tumors and SCAN-B TNBC cohorts.

Normal breast tissue and sorted immune cell epigenetic cohorts
Illumina 450K DNA methylation profiles for 96 normal breast tissue samples obtained from mastectomies, breast reductions, and prophylactic tissue were obtained from Gene Expression Omnibus (GEO) under accession number GSE67919 as processed beta values. Illumina 450K DNA methylation profiles (n=60) for 10 different blood cell types/fractions were obtained from Gene Expression Omnibus (GEO) under accession number GSE35069 as processed beta values.

Public cell line data
Genome coordinate conversion to hg19 for EPIC probes was carried out using the V1-version of "EPIC.hg19.manifest.tsv.gz" available through the Zhou-lab GitHub page (https://github.com/zhou-lab) and used instead of hg38 annotations where needed. Additional H3K27Ac bigWig tracks on hg38 for CD4, CD8, Macrophage and Breast luminal progenitor cells were obtained and processed as described in the Supplementary Methods. RNAseq data and matched Illumina 450K DNA methylation profiles for 34 TNBC cell lines were obtained from the study by Jovanovic et al. as processed FPKM expression values and beta values (n=372551 CpGs) (GEO GSE202770), respectively. Single cell RNAseq (scRNAseq) data for four of the 8 TNBC cell lines included in our study (HCC1937, MDA-MB-231, BT-549, and SUM185PE) were also obtained from Jovanovic et al. and processed as described (Supplementary Methods). A cancer cell line set comprising 869 cell lines of different malignancies with matched processed Affymetrix mRNA expression and Illumina 450K DNA methylation profiles were collected from Iorio et al. (GSE68379).

Survival analyses
Survival analyses were performed in R v4.2.2 using the survival package v3.5.8 with distant relapse-free interval (DRFI) as endpoint defined according to the STEEP guidelines. Survival curves were compared using Kaplan-Meier estimates and the log-rank test. Full details on the exclusion criteria for outcome analysis and individual patient treatment are available in Staaf et al. Nat Med 2019.

Statistical analyses
Calculated p-values are two-sided if not explicitly stated otherwise. If not stated otherwise Boxplot elements correspond to: (i) center line = median, (ii) box limits = upper and lower quartiles, (iii) whiskers = 1.5x interquartile range.

For manuscripts utilizing custom algorithms or software that are central to the research but not yet described in published literature, software must be made available to editors and reviewers. We strongly encourage code deposition in a community repository (e.g. GitHub). See the Nature Portfolio guidelines for submitting code & software for further information.

Data

Policy information about availability of data

All manuscripts must include a data availability statement. This statement should provide the following information, where applicable:

- Accession codes, unique identifiers, or web links for publicly available datasets
- A description of any restrictions on data availability
- For clinical datasets or third party data, please ensure that the statement adheres to our policy

The previously reported SCAN-B WGS data from Staaf et al. 6 used in this study are available from [https://data.mendeley.com/datasets/2mn4ctdpxp/3]. The previously reported SCAN-B RNA-sequencing data from Staaf et al. 70 used in this study are available from [https://data.mendeley.com/datasets/yzxtxn4nmd/3]. The DNA methylation data generated in this study for the SCAN-B discovery cohort have been deposited in the Gene Expression Omnibus database under accession code GSE148748 [https://www.ncbi.nlm.nih.gov/geo/query/acc.cgi?acc=GSE148748] and GSE148906 [https://www.ncbi.nlm.nih.gov/geo/query/acc.cgi?acc=GSE148906]. The DNA methylation data generated in this study for the SCAN-B validation cohort have been deposited in the Gene Expression Omnibus database under accession code GSE290981 [https://www.ncbi.nlm.nih.gov/geo/query/acc.cgi?acc=GSE290981]. Raw RNA sequencing data and ATAC-seq data

generated in this study for eight TNBC cell lines are available through the SRA archive at NCBI under BioProject accession PRJNA1189708 and study SRP547133 [https://www.ncbi.nlm.nih.gov/sra/?term=SRP547133]. The DNA methylation data generated in this study for the eight TNBC cell lines have been deposited in the Gene Expression Omnibus database under accession code GSE282347 [https://www.ncbi.nlm.nih.gov/geo/query/acc.cgi?acc=GSE282347]. Raw and normalized proteomic data generated in this study for eight TNBC cell lines are available in Supplementary Data 5, and mass spectrometry data have been deposited to the ProteomeXchange Consortium via the JPOST partner repository with the data set identifier PXD058472 [https://proteomecentral.proteomexchange.org/cgi/GetDataset?ID=PXD058472]. The previously reported breast cancer TCGA data used in this study is available from the GDC data portal [https://portal.gdc.cancer.gov]. The previously reported Iorio et al. 93 cancer cell line data used in this study are available from [https://www.ncbi.nlm.nih.gov/geo/query/acc.cgi?acc=GSE68379]. The previously reported normal breast tissue DNA methylation data from Hair et al. 87 used in this study are available from [https://www.ncbi.nlm.nih.gov/geo/query/acc.cgi?acc=GSE67919]. The previously reported gene expression and DNA methylation data from Jovanovic et al. 91 used in this study are available from [https://www.ncbi.nlm.nih.gov/geo/query/acc.cgi?acc=GSE202770]. The previously reported single cell RNAseq data for TNBC cell lines from Jovanovic et al. 91 used in this study are available from [https://www.ncbi.nlm.nih.gov/geo/query/acc.cgi?acc=GSE202771]. The previously reported sorted immune cell DNA methylation data from Reinius et al. 88 used in this study are available from [https://www.ncbi.nlm.nih.gov/geo/query/acc.cgi?acc=GSE35069]. Source data are provided with this paper. The remaining data are available within the Article, Supplementary Information or Source Data file.

Research involving human participants, their data, or biological material

Policy information about studies with human participants or human data. See also policy information about sex, gender (identity/presentation), and sexual orientation and race, ethnicity and racism.

Reporting on sex and gender	Sex and gender was not considered in the study design. Gender/sex has not been included in any analyses. Gender was not considered in the study design, as all patients were female. Gender of participants was determined based on self-report.
Reporting on race, ethnicity, or other socially relevant groupings	Not relevant as information was not available for any of the patient sets used.
Population characteristics	This study uses publicly deposited data associated with the SCAN-B (Staaf et al. Nat Med 2019 and NPJ Breast Cancer 2022) study and the TCGA cohort. Population details and characteristics are described in original studies.<br><br>Patients recruited to the SCAN-B observational cohort, on which study is based, is recruited in a population-based setting. Approximately 85-90% of ALL breast cancer patients with primary disease is enrolled in SCAN-B at the active sites (see Ryden et al. Br J Surg 105, e158-e168 (2018)). This means that there is no real cohort bias, and that research cohorts can be shown to mimic actual population-based cohorts. This population representativity has been demonstrated in, e.g., the study by Ryden et al.<br><br>The covariate-relevant population characteristics of the human research participants were not applicable to this study as patients were selected based on if there tumors had the molecular phenotypes of interest (requiring appropriate omics data).
Recruitment	Not relevant for the TCGA study as our usage is based on data deposited in GDC Data portal.<br><br>Patients were recruited to the SCAN-B observational cohort as described in previous studies (Ryden et al. Br J Surg 105, e158-e168 (2018). Selection of patients for this study was based on a defined SCAN-B data study including patients with RNAseq and WGS enrolled between between September 2010 and March 2015 reported by Staaf et al. Nat Med 2019. This study outlines the full characteristics, clinical registry data collection, end point definitions, WGS analysis etc.<br><br>Selection of patients for the validation cohort study based on a defined SCAN-B data release including patients with RNAseq enrolled between between September 1 2010 and May 31 2018 reported by Staaf et al. NPJ Breast Cancer 2022. This study outlines the full characteristics, clinical registry data collection, end point definitions, RNA-sequencing, etc.<br><br>There was no participant compensation for SCAN-B.
Ethics oversight	Ethical oversight was only applicable to SCAN-B as all other cohorts are publicly available and for which relevant ethical considerations have already been described.<br><br>Ethical approval was given for the SCAN-B study by the Regional Ethical Review Board in Lund, Sweden, governed by the Swedish Ethical Review Authority, Box 2110, 750 02 Uppsala, Sweden.<br><br>Inclusion and Ethics statement<br>Patients were enrolled in the Sweden Cancerome Analysis Network – Breast (SCAN-B) study (ClinicalTrials.gov ID NCT02306096) 21, 22 approved by the Regional Ethical Review Board in Lund, Sweden (registration numbers 2009/658, 2010/383, 2012/58, 2013/459, 2014/521, 2015/277, 2016/541, 2016/742, 2016/944, 2018/267 and the Swedish Ethical Review Authority (registration numbers 2019-01252, 2024-02040-02), governed by the Swedish Ethical Review Authority, Box 2110, 750 02 Uppsala, Sweden. All patients provided written informed consent prior to enrolment. All analyses were performed in accordance with patient consent and ethical regulations and decisions.

Note that full information on the approval of the study protocol must also be provided in the manuscript.

Field-specific reporting

Life sciences study design

All studies must disclose on these points even when the disclosure is negative.

Sample size	The discovery cohort is based on the cohort and publicly deposited data associated with the SCAN-B (Staaf et al. Nat Med 2019) study. As such, sample sizes are fixed.<br><br>No statistical method was used to predetermine sample size.<br><br>This study uses publicly deposited patient tumor data associated with the SCAN-B (Staaf et al. Nat Med 2019 and NPJ Breast Cancer 2022) and TCGA studies. As such, sample sizes are fixed. SCAN-B represented the primary discovery cohort due to its size. TCGA was included to portray specific methylation patterns in a general breast cancer population due its multi-omic nature (available DNA methylation and RNA-sequencing data).<br>GSE67919 was included as a normal breast tissue DNA methylation cohort as it was readily available from GEO. GSE68379 was included as a source of matched mRNA and DNA methylation data for pan-cancer cell lines as it represents the likely largest pan-cancer cell line cohort publicly available. GSE202770 was included as a source of matched mRNA, DNA methylation, and single-cell RNA-sequencing data for breast cancer cell lines as it represents the likely largest pan-cancer cell line cohort publicly available.
Data exclusions	All patients available from the reported SCAN-B cohort by Staaf et al. Nat Med 2019 was used that fitted the defined breast cancer subgroup (TNBC). For the validation cohort, we selected patients based on clinical subgroup (TNBC), available DNA, and enrollment in the same healthcare region as the discovery cohort. No data (patients) were excluded from the analyses.
Replication	No replication of WGS, RNAseq or DNA methylation analyses were performed for patient tumor samples. Proteomic analyses were performed in replicates using two biological cell line replicates. Results based on computational analyses of publicly available data or experimentally derived data in a primary discovery cohort (SCAN-B) was instead validated in independent cohorts for which public or novel data existed/was created. The latter included both data based on primary tumor tissue as well as in vitro cultured breast cancer cell lines.
Randomization	The experiments were not randomized. Randomization was not applicable to this study. The SCAN-B study population used in the current study originates from an ongoing population-based observational cohort study in south Sweden (SCAN-B, see ClinicalTrials.gov ID NCT02306096 and Ryden et al. Br J Surg 105, e158-e168 (2018)).<br><br>Patients were not randomized into any groups. Group definitions used were based on clinicopathological variables or molecular variables (like PAM50 subtypes, or derived epitypes).
Blinding	Blinding was not applicable to this study. This study is based on defined groups for which statistical comparisons were performed. There was no randomizations or different patient treatment arms etc. All studies used are retrospective cohorts. The sequencing facilities were however blinded to the patient and group id, but once data was collected and processed by the investigators blinding was not applicable.Patients were not randomized into any groups. Group definitions used were based on clinicopathological variables or molecular variables (like PAM50 subtypes).The Investigators were not blinded to allocation during experiments and outcome assessment.

Reporting for specific materials, systems and methods

We require information from authors about some types of materials, experimental systems and methods used in many studies. Here, indicate whether each material, system or method listed is relevant to your study. If you are not sure if a list item applies to your research, read the appropriate section before selecting a response.

Materials & experimental systems

n/a	Involved in the study	
x		Antibodies
	x	Eukaryotic cell lines
x		Palaeontology and archaeology
x		Animals and other organisms
	x	Clinical data
x		Dual use research of concern
x		Plants

Methods

n/a	Involved in the study	
x		ChIP-seq
x		Flow cytometry
x		MRI-based neuroimaging

Eukaryotic cell lines

Policy information about cell lines and Sex and Gender in Research

Cell line source(s)	Cell lines were obtained from commercial (ATCC) or in-house biobanks.
Authentication	All cell lines were sent for short tandem repeat (STR) profiling (Human cell line authentication service, Eurofins Genomics) to confirm cell line origin.
Mycoplasma contamination	All cell lines were sent for mycoplasma testing (Mycoplasmacheck service, Eurofin Genomics) and confirmed as mycoplasma free;
Commonly misidentified lines (See ICLAC register)	Not applicable

Clinical data

Policy information about clinical studies

All manuscripts should comply with the ICMJEguidelines for publication of clinical research and a completedCONSORT checklist must be included with all submissions.

Clinical trial registration	The SCAN-B study is a prospective observational study registered as ClinicalTrials.gov ID NCT02306096. As such it is not a phase 2 / 3 clinical trial. The TCGA study is not a registered trial.
Study protocol	SCAN-B is an observational cohort study. Thus there is no specific study protocol related to patient therapy as study inclusion does not affect patient therapy in any way.
Data collection	SCAN-B discovery cohort patients were recruited between September 2010 to March 2015 in the Southern Swedish Healthcare region, Sweden. Clinical data was obtained from the deposited data associated with the original SCAN-B study (Staaf et al Nat Med 2019).<br><br>For TCGA clinical data was obtained from online deposited data through the GDC portal.
Outcomes	Outcomes for all patients were obtained from the deposited data associated with the original SCAN-B studies. The full definition of clinical endpoints in SCAN-B patients are provided in the original study by Staaf et al. Nat Med 2019. In survival analyses the endpoints used is clearly stated.

Plants

Seed stocks	Not applicable
Novel plant genotypes	Not applicable
Authentication	Not applicable
